# In Silico-Based Discovery of Natural Anthraquinones with Potential against Multidrug-Resistant *E. coli*

**DOI:** 10.3390/ph15010086

**Published:** 2022-01-11

**Authors:** Hani A. Alhadrami, Wesam H. Abdulaal, Hossam M. Hassan, Nabil A. Alhakamy, Ahmed M. Sayed

**Affiliations:** 1 Department of Medical Laboratory Technology, Faculty of Applied Medical Sciences, King Abdulaziz University, P.O. Box 80402, Jeddah 21589, Saudi Arabia; hanialhadrami@kau.edu.sa; 2 Molecular Diagnostic Lab, King Abdulaziz University Hospital, King Abdulaziz University, P.O. Box 80402, Jeddah 21589, Saudi Arabia; 3 Special Infectious Agent Unit, King Fahd Medical Research Center, King Abdulaziz University, P.O. Box 80402, Jeddah 21589, Saudi Arabia; 4 Cancer and Mutagenesis Unit, Department of Biochemistry, Faculty of Science, King Fahd Medical Research Center, King Abdulaziz University, P.O. Box 80402, Jeddah 21589, Saudi Arabia; whabdulaal@kau.edu.sa; 5 Department of Pharmacognosy, Faculty of Pharmacy, Nahda University, Beni-Suef 62513, Egypt; 6 Department of Pharmacognosy, Faculty of Pharmacy, Beni-Suef University, Beni-Suef 62511, Egypt; 7 Department of Pharmaceutics, Faculty of Pharmacy, King Abdulaziz University, P.O. Box 80402, Jeddah 21589, Saudi Arabia; nalhakamy@kau.edu.sa

**Keywords:** *E. coli*, Ddl-B, Gyr-B, virtual screening, anthraquinone, AfroDb

## Abstract

*E. coli* is a Gram-negative bacterium that causes different human infections. Additionally, it resists common antibiotics due to its outer protective membrane. Natural products have been proven to be efficient antibiotics. However, plant natural products are far less explored in this regard. Accordingly, over 16,000 structures covering almost all African medicinal plants in AfroDb in a structural-based virtual screening were used to find efficient anti-*E. coli* candidates. These drug-like structures were docked into the active sites of two important molecular targets (i.e., *E. coli*’s Ddl-B and Gyr-B). The top-scoring hits (i.e., got docking scores < −10 kcal/mol) produced in the initial virtual screening (0.15% of the database structures for Ddl-B and 0.17% of the database structures for Gyr-B in the database) were further refined using molecular dynamic simulation-based binding free energy (Δ*G*) calculation. Anthraquinones were found to prevail among the retrieved hits. Accordingly, readily available anthraquinone derivatives (10 hits) were selected, prepared, and tested in vitro against Ddl-B, Gyr-B, multidrug-resistant (MDR) *E. coli*, MRSA, and VRSA. A number of the tested derivatives demonstrated strong micromolar enzyme inhibition and antibacterial activity against *E. coli*, MRSA, and VRSA, with MIC values ranging from 2 to 64 µg/mL. Moreover, both *E. coli*’s Ddl-B and Gyr-B were inhibited by emodin and chrysophanol with IC_50_ values comparable to the reference inhibitors (IC_50_ = 216 ± 5.6, 236 ± 8.9 and 0.81 ± 0.3, 1.5 ± 0.5 µM for Ddl-B and Gyr-B, respectively). All of the active antibacterial anthraquinone hits showed low to moderate cellular cytotoxicity (CC_50_ > 50 µM) against human normal fibroblasts (WI-38). Furthermore, molecular dynamic simulation (MDS) experiments were carried out to reveal the binding modes of these inhibitors inside the active site of each enzyme. The findings presented in this study are regarded as a significant step toward developing novel antibacterial agents against MDR strains.

## 1. Introduction

The emergence of drug-resistant bacterial variants is the main driving force behind the discovery of novel antimicrobial agents. The enzymatic system responsible for the synthesis of peptidoglycan, a key bacterial cell wall component that provides structural integrity to enable bacteria to resist internal osmotic pressure, is a validated target for antibacterial therapeutics [1,2,3]. Unlike the final stages of peptidoglycan construction suppressed by β-lactam and glycopeptide antibiotics, the early stages of peptidoglycan biosynthesis have drawn limited attention as possible therapeutic targets.

To date, there are only two drugs that can inhibit the peptidoglycan synthesis intracellularly: fosfomycin, an inhibitor of MurA ligase, the enzyme that adds phosphoenolpyruvate to UDP-N-acetylglucosamine in the initial step to synthesize the cell wall precursor UDP-N-acetylmuramic acid. The second drug is D-cycloserine, an inhibitor of alanine racemase and D-alanine-D-alanine ligase (Ddl-B), an enzyme that binds D-alanine to another amino acid [4,5]. Mur ligases, such as MurC, MurD, MurE, and MurF, combine L-Ala, D-Glu, m-Dpm or L-Lys, and D-Ala-D-Ala to form the final intracellular peptidoglycan precursor UDPMurNAc-pentapeptide. Ddl-B is responsible for providing D-alanyl-D-alanine, the MurF substrate. Since peptidoglycan chains cross-linking occurs between the C6 – NH_2_-group of meso-diaminopimelic acid (Gram-negative) or the NH_2_ group of pentaglycine (Gram-positive) and the penultimate D-Ala in a second pentapeptide strand, this terminal dipeptide (i.e., D-alanyl-D-alanine) has a critical role in the assembly of the bacterial cell wall [6,7].

Conversely, DNA gyrase is a type II topoisomerase enzyme necessary for maintaining topology and integrity during bacterial DNA replication and transcription [1]. It comprises four subunits (two As and two Bs) that are joined to produce a tetrameric holoenzyme [8]. The enzyme Gyr-A subunits bind to the DNA, and the Gyr-B subunits hydrolyze two ATP molecules to provide the whole enzyme complex with the energy required to relax DNA supercoils. DNA gyrase is considered one of the main antibacterial targets that have been validated clinically in many bacteria. Fluoroquinolones are known DNA gyrase poisons that selectively target the enzyme A subunit, making it toxic to the bacterial cell. Over the past 20 years, they have been successful as antibacterial agents. However, many resistant strains have recently emerged [9]. Accordingly, different research groups worldwide are working on the discovery of new antibacterial agents that target the less explored DNA gyrase B subunits [10,11]. Structure-based virtual screening is broadly applied for hit identification and could also be utilized for lead optimization. The docking, scoring, and ranking of different molecules are part of the virtual screening process to discover novel enzyme inhibitors. With the aid of other physics-based molecular modeling tools (e.g., molecular dynamic simulation; MDS), it becomes possible to uncover the mode of action of lead compounds, indicating how they can be structurally optimized to be more potent. During the past 15 years, computer-aided drug design (CADD) has played a significant role in the development of novel antibacterial therapeutics [1,2,3].

Natural products have a bright history in fighting pathogenic microbes, including bacteria, and provided humanity with many precious antibiotics (e.g., penicillins, macrolides, and aminoglycosides) that have saved many lives. Additionally, greener methods can usually produce natural products (e.g., extraction, genetic engineering, and fermentation) compared to synthetic compounds [12]. Medicinal plants are a huge reservoir of bioactive molecules. However, their potential as antibacterial agents is under-explored [13]. Despite the unmatched molecular diversity of plant natural products, almost all currently available antibiotics are of microbial origin [14].

Recently, a comprehensive investigation of plant natural products was initiated to discover potential antibacterial leads [2,3]. Herein, these efforts were continued to explore medicinal plants, particularly those present in North Africa, aiming to find potential antibacterial agents, particularly against *E. coli*, that can target either Ddl-B or Gyr-B, or both. Accordingly, the crystal structures of *E. coli* Ddl-B and Gyr-B were used to initiate a structural-based virtual screening of all plant-derived natural products hosted in AfroDb [15]. This well-curated database contains > 16,000 plant-based natural products recovered from African medicinal plants and possesses good calculated ADMET properties [15].

## 2. Results and Discussion

### 2.1. Molecular Docking-Based Virtual Screening

Two Ddl isoforms, Ddl-A and Ddl-B, occur in *E. coli*, with identical kinetic properties and substrate selectivity. The projected amino acid sequences of *E. coli* Ddl-A and Ddl-B were aligned to reveal roughly 35% similarity for both enzymes. Ddl-B is well-characterized in literature and has many more crystal structures in Protein Data Bank (PDB) than its counterpart Ddl-A. Hence, it was selected for docking experiments along with the *E. coli* Gyr-B (PDB code: 2DLN and 6KZV, respectively) [16,17].

**Figure 1:**
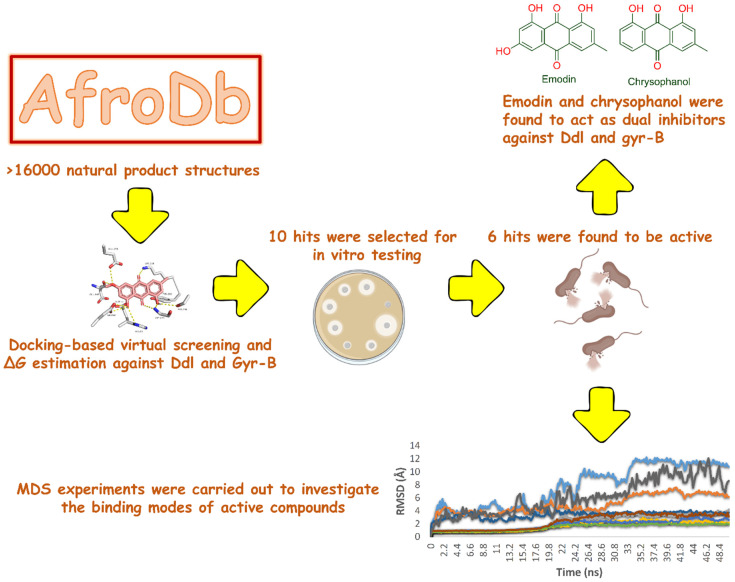
The workflow of the present study.

Findings obtained from the present investigation are summarized in Figure 1. First, the co-crystallized ligands were re-docked into the active site of Ddl-B or Gyr-B. It was found that the conformations of the re-docked ligands were perfectly superimposed on the co-crystallized ligands with RMSD values of 0.836 Å and 0.913 Å, respectively. This validated docking protocol was then used for docking-based screening of AfroDb (http://african-compounds.org/about/afrodb/ (accessed on 2 October 2021); 16,359 compounds).

**Figure 2:**
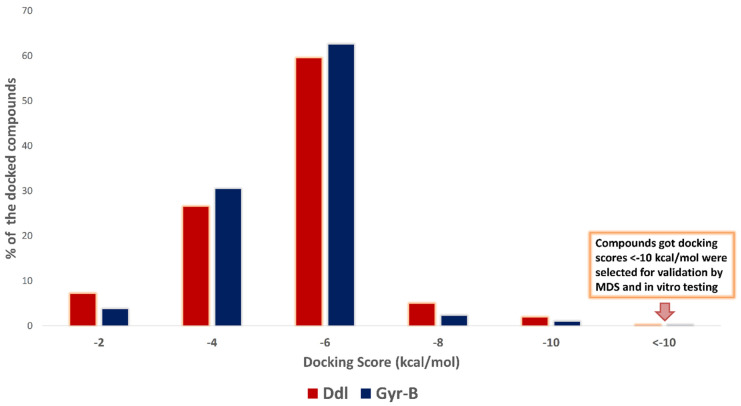
Score distribution of the docked compounds in AfroDb against Ddl-B and Gyr-B.

Docking results against each protein revealed that ~5% of the compounds got docking scores > −2 kcal/mol, ~28% got scores between −2 and −4 kcal/mol, ~60% got scores between −4 and −6 kcal/mol, and ~3% got scores between −6 and −8. Only about 1.4% of the docked compounds against both proteins got scores < −8 kcal/mol (Figure 2). A cut-off score of −10 kcal/mol was used to select the top-scoring hits confidently. So, 24 compounds (0.15% of the database compound list) were selected for Ddl-B and 27 compounds (0.17% in the database) for Gyr-B. Subsequently, these top-scoring hits were subjected to MDS-based Δ*G* estimation using the free energy perturbation (FEP) method [18] to validate their binding efficiency with the corresponding active sites. The docking scores of top-scoring hits in each target along with their estimated Δ*G* values are listed in Tables S1 and S2.

**Figure 3:**
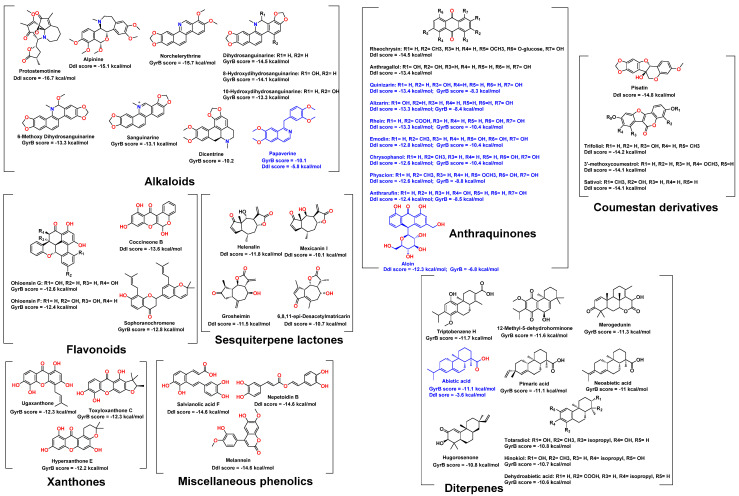
Top-scoring compounds retrieved from docking-based screening of AfroDb against *E. coli* Ddl-B and Gyr-B. The compounds were grouped according to their chemical classes and the docking scores against either Ddl-B or Gyr-B were presented under each compound (Ddl-B scores or Gyr-B scores, respectively). Blue-colored compounds were selected for in vitro testing.

As shown in Figure 3, 24 compounds belonging to different chemical classes got docking scores < −10 kcal/mol upon docking-based virtual screening against *E. coli* Ddl-B. They comprised ten anthraquinones, four sesquiterpenes, three coumestan derivatives, two alkaloids, two phenolic acids, one lignan, one dihydrobenzofuran, and one isoflavone. These hits got Δ*G* values < −7 kcal/mol (Table S1), indicating that they were potential ligands for the Ddl-B active site. Anthraquinone derivatives were the most prevalent compounds among the Ddl-B top-scoring hits (ten compounds). Moreover, their Δ*G* values (~−8.5 kcal/mol) were comparable to that of the Ddl-B co-crystallized ligand (−8.9 kcal/mol). Accordingly, eight derivatives out of these anthraquinones (compounds 13–20) were selected for in vitro testing as they were easily accessed from their plant sources.

Regarding Gyr-B, 27 compounds from five chemical classes were retrieved as the top-scoring hits (Figure 3). They were classified as nine abietane-type diterpenes, eight alkaloids, three xanthones, three flavonoids, and three anthraquinones. All of these hits got Δ*G* values < −7 kcal/mol (Table S2), except for ugaxanthone (10), dicentrine (26), and papaverine (27), got average Δ*G* values of −6.5 kcal/mol.

The most prevailing compounds among the Gyr-B top-scoring hits were Abietane-type diterpenes and isoquinoline alkaloids. However, they were not accessible from their plant sources, or their plant sources were not available in Egypt, except for abietic acid (16), which is considered the main constituent in the common medicinal resin, colophony [19]. Hence, abietic acid (16) was selected for in vitro testing to represent this class of compounds. Moreover, the three anthraquinones, emodin, physcion, and rhein, were also found to be top hits for Ddl-B, and thus they were also selected for in vitro testing along with the lowest scoring hit in this list, papaverine which is highly abundant in its plant source (i.e., *Papaver somniferum*) [20].

### 2.2. In Vitro Validation

The selected hits for in vitro testing were either isolated and purified (~98% purity) from their plant sources or purchased for maximum purity. Thereafter, they were tested for their antibacterial potential and inhibitory activity against *E. coli* Ddl-B and Gyr-B. As shown in Table 1, selected top-scoring hits against Ddl-B and Gyr-B showed varying in vitro results. Quinizarin and alizarin moderately inhibited the activity of both Ddl-B and Gyr-B in vitro. Moreover, quinizarin showed moderate activity against vancomycin-resistant *S. aureus* (VRSA) (MIC 16 µg/mL), while alizarin was inactive (MIC > 64 µg/mL). Both compounds showed weak activities against *E. coli* and methicillin-resistant *S. aureus* (MRSA) (MIC 64 µg/mL).

Rhein, emodin, chrysophanol, physcion were more potent against Ddl-B than the reference Ddl-B inhibitor D-cycloserine. Furthermore, emodin and chrysophanol were active against Gyr-B with inhibitory activity comparable to novobiocin, the reference Gyr-B inhibitor (IC_50_ 0.81 and 1.5 µM, respectively). They were active against most of the tested bacterial strains with MIC values 2–4 µg/mL. Hence, emodin and chrysophanol are considered promising lead antibacterial compounds for future development as Ddl-B and Gyr-B dual inhibitors. Previously, emodin, physcion, and chrysophanol have shown antibacterial potential against a wide spectrum of bacterial species. However, their exact mode of action is still elusive [21,22,23,24]. Anthrarufin showed moderate activity against Gyr-B, but its activity against Ddl-B was comparable to the reference inhibitor D-cycloserine. It showed very good antibacterial activity against both *E. coli* and MRSA (MIC 2 µg/mL) but was inactive against VRSA (MIC > 64 µg/mL).

The glycosylated anthraquinone derivative aloin got a good docking score and Δ*G* value, particularly with Ddl-B. However, experimentally it exhibited weak inhibition against both enzymes (i.e., Ddl-B and Gyr-B). Moreover, it was inactive against all tested bacteria (MIC > 64 µg/mL). These odd results may be attributed to the poor drug-like properties of aloin (i.e., has more than 5 NH or OH groups and topological polar surface area >140 Å^2^).

All the previous compounds selected from docking-based screening showed comparable Δ*G* values and in vitro results were of the same structural class (i.e., anthraquinone derivatives). Accordingly, the unsubstituted anthraquinone scaffold was also tested and was inactive against both enzymes and all tested bacteria. Based on the previous discussion, hydroxylation at both rings A and C seemed essential to produce antibacterial derivatives, particularly against *E. coli*. It is worth noting that this class of compounds (i.e., anthraquinones) is structurally different from the reference inhibitors D-cycloserine and novobiocine. Hence, they offer a new scaffold for the development of a new series of inhibitors against both Ddl-B and Gyr-B.

Regarding abietic acid, which was among the selected top-scoring hits for Gyr-B, it showed potent inhibitory activity against Gyr-B (IC_50_ 1.4 µM) and was inactive against Ddl-B. It showed moderate activity against the tested bacterial strains (MIC 16–64 µg/mL). Previous reports have indicated that abietic acid and other abietane-type diterpenoids have interesting antimicrobial properties against many pathogenic bacteria [25,26]. Papaverine got a good docking score against Gyr-B. However, its Δ*G* negative value was considerably low. The in vitro results of papaverine against both enzymes were consistent with the in-silico results and inactive against all tested bacteria (Table 1).

To evaluate the cellular toxicity of the active hits (Table 2), they were tested in vitro against the human normal fibroblast cell line WI-38. Rhein, physcion, and abietic acid showed the lowest toxicity towards WI-38 cell lines (CC_50_ > 50 µM). In contrast, emodin, chrysophanol, anthrarufin, and quinizarin showed moderate cellular toxicity (CC_50_ = 49.23 ± 1.39, 48.15 ± 1.39, 29.67 ± 0.76, and 20.11 ± 0.56 µM, respectively), which was comparable to the anticancer drug doxorubicin.

### 2.3. Molecular Modeling

Most of the selected top-scoring hits showed positive in vitro results. To further uncover the mode of interaction of these compounds with both Ddl-B and Gyr-B, 50 ns MDS experiments were performed, and the last snapshots of the MDS trajectories were analyzed.

**Figure 4:**
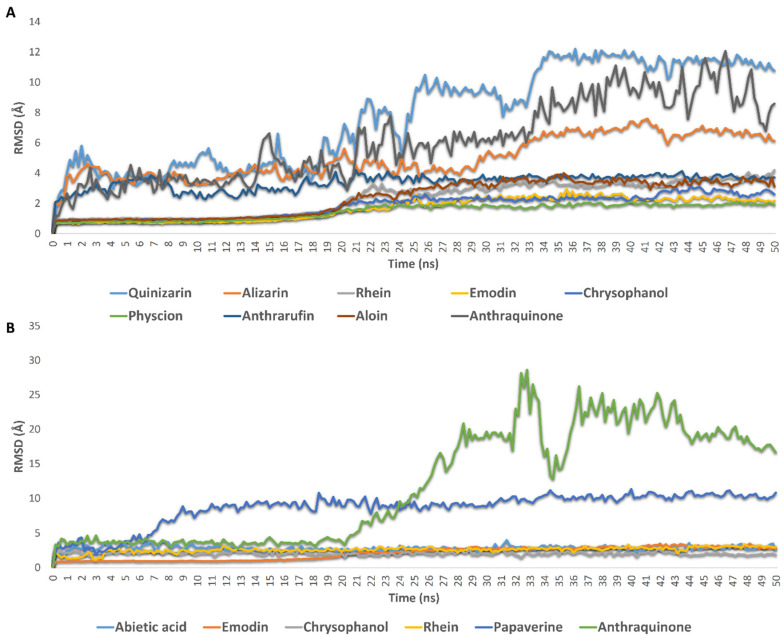
RMSDs of active top-scoring hits against inside the active site of Ddl-B and Gyr-B (**A**,**B**, respectively). RMSD of the unsubstituted anthraquinone was also studied to highlight the role of hydroxylation in the stability of this type of compounds.

First, Ddl-B selected top-scoring compounds (Figure 3): As shown in Figure 4A, root mean square deviations (RMSDs) of rhein, emodin, chrysophanol, physcion, anthrarufin, and aloin clustered together at ~2.6 Å, indicating that they established stable binding with Ddl-B active site over the simulation time with low deviations from the starting binding orientations (i.e., the docking poses). Such binding stability could be linked to the hydroxylation pattern of these anthraquinone derivatives (i.e., the hydroxylation at both rings A and C). Quinizarin, alizarin, and the unsubstituted anthraquinone clustered separately at higher RMSDs (~7.1 Å), indicating significant instability over the simulation time. These findings are in good accordance with the in vitro results, except for aloin which was stable inside the Ddl-B active site according to the modeling study (Average RMSD = 2.1 Å), however, it was inactive in vitro. This could be attributed to its sugar moiety that probably hinders its entrance into the active site in the first place.

**Figure 5:**
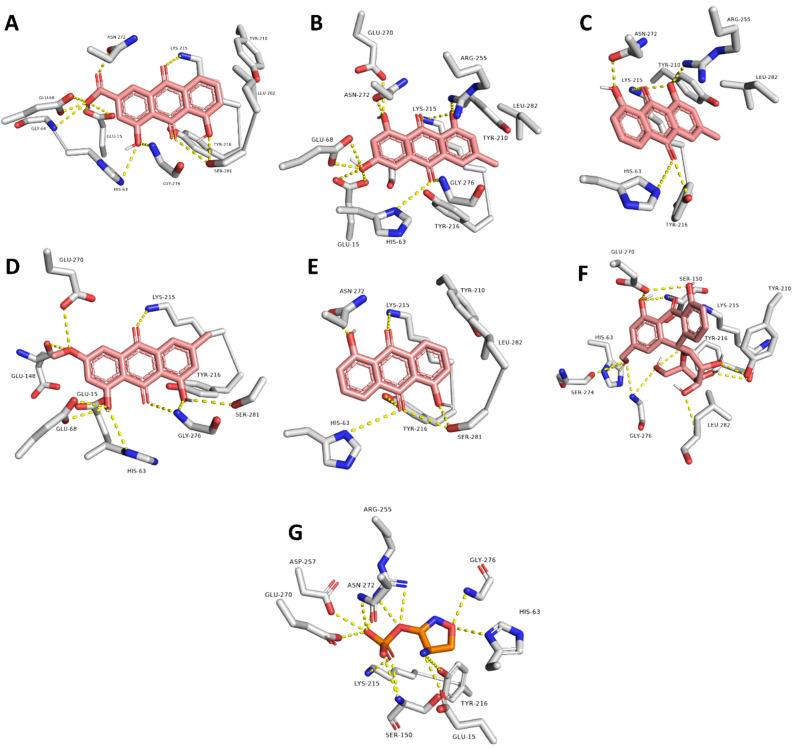
Binding modes of the active top-scoring hits (i.e., rhein, emodin, chrysophanol, physcion, anthrarufin, aloin; brick red-colored structures; (**A**–**F**), respectively) the co-crystalized ligand, D-cycloserine (orange-colored structure; (**G**)) inside the active site of Ddl-B. These binding modes were derived from the MDS experiments (i.e., the last snapshots of the 50 ns trajectories).

The last snapshots of rhein, emodin, chrysophanol, physcion, anthrarufin, and aloin MDS showed that they interacted with amino acid residues of the active site through multiple H-bonding, particularly with HIS-63, GLY-64, GLU-68, LYS-215, TYR-216, ASN-272, and SER-281 (Table 3 and Figure 5).

**Figure 6:**
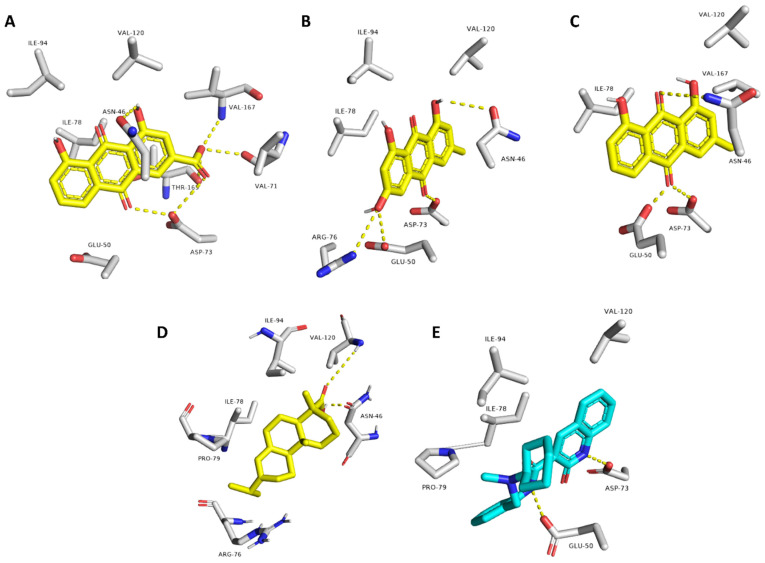
Binding modes of the active top-scoring hits (i.e., rhein, emodin, chrysophanol and abietic acid; yellow-colored structures; (**A**–**D**), respectively) and the co-crystalized ligand, 2-oxo-1,2-dihydroquinoline (cyan-colored structure; (**E**)) inside the ATPase site of Gyr-B. These binding modes were derived from the MDS experiments (i.e., the last snapshots of the 50 ns trajectories).

Second, Gyr-B selected top-scoring compounds (Figure 3): The RMSDs of rhein, emodin, chrysophanol, and abietic acid clustered together at ~2 Å. Additionally, they showed low fluctuations throughout the simulation, indicating very good stability inside the Gyr-B ATPase site. In contrast, papaverine and the unsubstituted anthraquinone were far less stable inside the ATPase site (average RMSD = 8.6, 11.9 Å, respectively) (Figure 4B). The last snapshots derived from the MDS of these active compounds (Figure 6) revealed that they established a number of stable H-bonds with multiple amino acid residues inside the protein ATPase site, e.g., ASN-46, GLU-50, ASP-73 (Table 4). The stability of abietic acid was mediated by both H-bonding between its carboxylate moiety and ASN-46 and VAL-120, and hydrophobic interactions between the molecule hydrophobic body and ILE-78, PRO-79, and ILE-94.

## 3. Materials and Methods

### 3.1. Preparation of E. coli Target Proteins and the Compounds Dataset

The Protein Data Bank (http://www.pdb.org (accessed on: 23 September 2021)) was used to get crystal structures of *E. coli* Ddl-B and Gyr-B (PDB codes: 4C5A and 6KZV) [17,27]. All heteroatoms and water particles were eliminated for molecular modeling investigations. The calculated RMSD between the Gyr-B structure (i.e., the ATPase site) used in the present study (PDB codes: 6KZV) and its structure in a crystalized holoenzyme (PDB codes: 6RKS) was 0.16 Å. Accordingly, the structure of the isolated Gyr-B is almost the same as its structure in the holoenzyme complex.

The structures of the evaluated plant metabolites were obtained from the AfroDb (http://african-compounds.org/about/afrodb/ (accessed on, 2 September 2021)) [15] online collection, which resulted in a final combined dataset of 16,359 compounds.

### 3.2. Docking-Based Virtual Screening

PyRx software [28] was used for the docking-based screening of all structures in the AfroD. This structure-based screening software used AutoDock Vina as a docking machine [29]. The docking protocol was validated by redocking the co-crystalized ligands into the binding sites of their corresponding proteins, where the resulted binding modes of the top poses were very close to that of the crystalized structures (RMSD = 1.1 Å for the Ddl-B’s co-crystallized ligand and RMSD = 0.97 Å for the Gyr-B’s co-crystallized ligand). Moreover, the used reference inhibitors were also docked into the active site of each protein for further validation (Table 1). Subsequently, all structures hosted in AfroDb were downloaded as SMILES codes and loaded to PyrX. After that, the structures were energy minimized and prepared for docking. Then, protein structures were loaded to the software and prepared for docking. The binding site was determined according to the enzyme co-crystallized ligand. The coordinates of the grid boxes were: x = 18.68; y = 8.47; z = 46.21, and x = −7.58; y = 16.39; z = 2.44 for Ddl-B and Gyr-B, respectively. The size of the grid boxes was set to be 10 Å. Exhaustiveness was set to be 24. Ten poses were generated for each docking experiment. Finally, the resulted docking scores were arranged and the best-scoring pose for each compound was selected. Docking poses were analyzed and visualized using Pymol software [29].

### 3.3. Drug-Likeness Analysis

The commercially available software LigandScout 4.3 was used to anticipate the examined compounds drug-like characteristics [30]. To execute the drug-likeness computations, a list of SMILES codes for the top-scoring compounds was generated and loaded into the software (e.g., molecular weight, hydrogen bond donors, hydrogen bond acceptors, number of rotatable bonds, topological polar surface area, and logP). Finally, the calculated parameters were checked for each compound according to Lipinski and Vebers’ drug similarity criterion.

### 3.4. Molecular Dynamic Simulation and Binding Free Energy Estimation

As previously described, binding free energy estimation (Δ*G*) and molecular dynamic simulations were performed [31,32,33]. These methods are described in detail in the supplementary file, pages S4, S5.

### 3.5. Compounds Preparation

The selected top-scoring hits (Table 1), along with reference antibiotics (i.e., D-cycloserine and novobiocin), were purchased (quinizarin, alizarin, anthrarufin, anthraquinone, rhein, papaverine and aloin) from Alfa Aesar, MA, USA and Sigma-Aldrich, Saint Louis, MO, USA or isolated (emodin, chrysophanol, physcion, and abietic acid) from their natural source following the previously described methods [34,35]. More details can be found in the supplementary file, pages S6 and S7. The identity and purity of the isolated compounds were checked by ^1^HNMR and LC-HRESIMS analysis to ensure > 95% purity. These isolated compounds were used for the preliminary biological evaluation only. The tested compounds were purchased for enzyme assays and MIC evaluations to ensure maximum possible purity (Alfa Aesar, Saint Louis, MO, USA).

### 3.6. In Vitro Testing

#### 3.6.1. Enzyme Assay

Ddl-B and Gyr-B activity assays were performed according to the manufacturer protocols (ProFoldin, Catalogue No. DDA100KE, and Inspiralis, Catalogue No. ATPG001, UK).

Ddl-B enzymatic activity assay is based on measuring the inorganic phosphate generated from the D-Alanine: D-Alanine ligation reaction. The inorganic phosphate is detected by absorbance at 650 nm. The assay reactions and detection were performed by using a 96-well plate. Different concentrations of the test compounds and the reference inhibitor D-cycloserine were prepared in DMSO (from 10 µM to 1000 µM). Hence, 50 µL of the test compound in DMSO was then added to 297 μL of premix composed of 261 μL of water, 33 μL of 10× buffer and 3.3 μL of 100× *E. coli* DAla:D-Ala ligase. Thereafter, 33 μL of 10× enzyme substrate was prepared by mixing 3.3 μL of 100× enzyme substrate with 29.7 μL of water. Finally, 27 μL of the premix with 3 μL of the 10× enzyme substrate in each well were mixed. After 60 min of incubation at 37 °C, 45 μL of MPA3000 dye was added into the 30 μL of the reaction mixture. After 5 min of incubation, absorbance was measured at 650 nm.

Gyr-B assay measured the capability of the tested compounds to hinder the ATPase activity of Gyr-B subunits. This assay links the hydrolysis of ATP by gyrase to the conversion of NADH to NAD^+^, which can be measured by a change in absorbance at 340 nm. The assay reactions and detection were performed using a 96-well plate. Different test compounds and the reference inhibitor novobiocin concentrations were prepared in DMSO (from 0.05 µM to 100 µM). First, the assay mix was prepared by mixing 20 μL of the assay buffer (50 mM Tris.HCl (pH 7.5), 1 mM EDTA, 5 mM magnesium chloride, 5 mM DTT, 10% (*w/v*) glycerol), 1 μL phosphoenolpyruvate (80 mM in water), 1.5 μL pyruvate kinase/lactate dehydrogenase stock, 2 μL NADH (20 mM in water), and 58.8 μL water. Thereafter, 63.3 μL of assay mix and 10 μL of the test compound solution and 20 μL of the enzyme (*E. coli* gyrase B43 domain; 20 μM concentration) were added into the wells and mixed by pipetting. After incubation for 10 min at 25 °C, 6.7 uL of ATP (30 mM) was added to each well. After 60 min of incubation at 25 °C, the absorbance was measured at 340 nm.

#### 3.6.2. Antibacterial Evaluation

The selected top-scoring hits were evaluated for their antibacterial effect against two clinical multidrug-resistant strains of enterotoxigenic *E. coli*. Both isolates were isolated as clinical samples from Beni Suef general hospital, Beni Suef, Egypt. *E. coli* MDR^a^ was found to be resistant to florquinolones, amikacin, and ß-lactam antibiotics, while *E. coli* MDR^b^ was resistant to ß-lactam antibiotics and several macrolides (Table S3, unpublished data). MRSA (ATCC 25529) and VRSA (clinical isolate from Beni Suef general hospital, Beni Suef, Egypt) were also used to further evaluate the tested compounds against resistant Gram-positive bacteria.

Minimum inhibitory concentrations for the test compounds were analyzed following the standard agar dilution method [36] using Meuller-Hinton (MH) agar medium against different Gram-negative and positive bacteria (Table 1). Firstly, the tested compounds were prepared as a stock suspension with a 10 mg/mL final concentration in DMSO. Subsequently, the prepared stocks were diluted in 10 mL molten soft agar (0.5% agar) to obtain a concentration range from 1–64 µg/mL, then mixed and poured over a surface of MH agar (2% agar) plates and left for solidification. Finally, exponentially grown (O.D 600 nm of 0.6) test strains (Table 1) were diluted to a final density of 10^6^ CFU/mL in MH broth and spotted (1 µL/spot) over the soft agar. Then, the plates were left for drying and incubated at 37 °C for 18–20 h. Controls were conducted using DMSO seeded soft agar. At the end of the experiment, the MIC for each compound was identified as the minimum concentration that inhibited the growth of the tested strain. The experiments were carried out in triplicates.

#### 3.6.3. Cellular Toxicity against Normal Fibroblasts WI-38

All selected top-scoring compounds were tested for their cellular toxicity against human fibroblasts cell line (WI-38). Cell line (WI-38; ATCC: CCL-75) was maintained in earth RPMI 1640 medium comprising 10% heat-inactivated fetal bovine serum (FBS) and grown at 37 °C and 5% CO_2_. The cell line was seeded on 96-well cell culture plates (2 × 10^4^ cells/well). Cells were stimulated with tested compounds at different concentrations (from 1 to 50 µM) overnight in triplicates. Doxorubicin was used as a positive control. Cell viability was assessed by MTT assay. To normalize cell viability values, each plate included a triplicate of untreated cells considered as 100% viability, and a triplicate of cells treated with a cytotoxic mixture (200 ng/mL TNF, 200 ng/mL CD95L, 200 ng/mL TRAIL, 5 μg/mL CHX, 1% (*w/v*) sodium azide 20%) considered as 0% viability. All other viability values were normalized according to the averages of these triplicates and analyzed by the Graph Pad Prism 5 software (La Jolla, CA, USA).

## 4. Conclusions

Herein, a library of natural products specific to African medicinal plants hosted in AfroDb was investigated to obtain antibacterial hits against MDR *E. coli*. More than 16,000 structures were docked against two essential and underexplored molecular targets (i.e., Ddl-B and Gyr-B). Top-scoring hits retrieved from this virtual screening were then subjected to MDS-based binding free energy (Δ*G*) estimation to further evaluate these hits’ binding efficiency. Readily available hits that were structurally related to each other were evaluated in vitro. A number of anthraquinone derivatives and abietic acid showed potent enzyme inhibitory and antibacterial activities against *E. coli* and two resistant Gram-positive bacteria (i.e., MRSA and VRSA). Emodin and chrysophanol were found to inhibit both Ddl-B and Gyr-B. All of the active compounds showed moderate cellular toxicity towards normal human fibroblasts cells (WI-38) that was to some extent comparable to anticancer drug doxorubicin. The findings presented in this study highlighted the efficiency of integrating different virtual screening approaches in facilitating the discovery of bioactive therapeutics. Additionally, it highlighted the potential of plant-based natural products as a promising source of antibacterial candidates. Accordingly, deeper investigation of this enormous source of natural products should be performed to support the discovery of potential antibiotics.

## Figures and Tables

**Table 1:** Docking scores, Δ*G*, IC_50_, *K*_i_, MIC values of top-scoring hits.

Compound	Docking Score		Δ*G*		IC_50_ (µM)		*K*_i_ (µM)		MIC (µg/mL)			
	Ddl-B	Gyr-B	Ddl-B	Gyr-B	Ddl-B	Gyr-B	Ddl-B	Gyr-B	*E. coli* (MDR) ^a^	*E. coli* (MDR) ^b^	MRSA	VRSA
Quinizarin	−13.4	−8.3	−9.1	−5.5	515 ± 8.3	38.8 ± 2.3	230 ± 8.3	17.9 ± 1.4	64	64	64	16
Alizarin	−13.3	−8.4	−8.5	−6.5	468 ± 6.5	17.9 ± 1.2	220 ± 4.3	10.4 ± 1.1	64	>64	64	>64
Rhein	−13.3	−10.4	−8.4	−9.1	281 ± 6.1	11.5 ± 1.2	133 ± 2.3	7.33 ± 0.9	8	2	>64	8
Emodin	−12.8	−10.4	−9.6	−9.9	216 ± 5.6	0.81 ± 0.3	102 ± 4.8	0.22 ± 0.1	2	2	2	2
Chrysophanol	−12.6	−10.4	−9.1	−9.3	236 ± 8.9	1.5 ± 0.5	105 ± 4.9	0.83 ± 0.2	4	4	>64	4
Physcion	−12.6	−8.8	−8.3	−6.9	244 ± 7.2	10.9 ± 0.5	119 ± 9.1	6.6 ± 0.9	2	2	2	2
Anthrarufin	−12.4	−8.5	−8.1	−7.7	350 ± 7.4	6.93 ± 1.6	345 ± 10.1	4.1 ± 1.6	2	2	2	>64
Aloin	−12.3	−6.8	−8.7	−5.2	927 ± 7.4	22.4 ± 2.1	529 ± 12.3	10.48 ± 1.6	>64	>64	>64	>64
Anthraquinone	−9.3	−7.8	−5.1	−5.6	>1000	>100	>1000	>1000	>64	>64	>64	64
Abietic acid	−3.6	−11.1	−1.7	−8.8	>1000	1.4 ± 0.4	>1000	0.77 ± 0.1	64	16	>64	16
Papaverine	−5.8	−10.1	−2.2	−6.2	>1000	>100	>1000	>1000	>64	>64	>64	>64
D-cycloserine *	−7.3	-	−7.8	-	362	-	118 ± 11.7	-	16	16	32	32
Novobiocin **	-	−15.3	-	−10.4	-	0.47	-	-	64	64	1	1

^a^ and ^b^ are multi-drug resistant *E. coli* clinical isolates. * The reference Ddl-B inhibitor. ** The reference Gyr-B inhibitor.

**Table 2:** CC_50_s of the selected compounds against WI-38 cell lines.

Compound	CC_50_ (µM)
Quinizarin	20.11 ± 0.56
Alizarin	25.89 ± 0.69
Rhein	>50
Emodin	49.23 ± 1.39
Chrysophanol	48.15 ± 1.39
Physcion	>50
Anthrarufin	29.67 ± 0.76
Aloin	34.74 ± 0.94
Abietic acid	>50
Papaverine	36.83 ± 0.93
Doxorubicin	26.18 ± 0.61

**Table 3:** Interactions of the active Ddl-B top-scoring compounds inside the enzyme active site.

Compound	Interaction	
	H-Bonding	Hydrophobic
Rhein	GLU-15, HIS-63, GLY-64, GLU-68, LYS-215, TYR-216, ASN-272, SER-281	LYS-215, LEU-282
Emodin	GLU-15, HIS-63, GLY-64, LYS-215, ARG-255, GLU-270, ASN-272, GLY-276	LYS-215, LEU-282
Chrysophanol	HIS-63, LYS-215, TYR-216, ARG-255	LYS-215, LEU-282
Physcion	GLU-15, HIS-63, GLY-64, GLU-68, GLU-148, GLY-276, SER-281	LYS-215
Anthrarufin	HIS-63, LYS-215, TYR-216, ASN-272, SER-281	LYS-215
Aloin	GLU-15, HIS-63, GLY-64, GLU-68, SER-150, LYS-215, TYR-216, ASN-272, LEU-282	-
Co-crystalized ligand (D-cycloserine)	GLU-15, HIS-63, GLY-64, SER-150, ARG-255, GLU-270, ASN-272, GLY-276	-

**Table 4:** Interactions of the active Gyr-B top-scoring compounds inside the enzyme’s ATPase site.

Compound	Interaction	
	H-Bonding	Hydrophobic
Rhein	ASN-46, VAL-71, ASP-73, VAL-167	ILE-78
Emodin	ASN-46, GLU-50, ASP-73, ARG-76	ILE-78
Chrysophanol	ASN-46, GLU-50, ASP-73	ILE-78
Abietic acid	ASN-46, VAL-120	ILE-78, PRO-79, ILE-94
Co-crystalized ligand (2-oxo-1,2-dihydroquinoline)	GLU-50, ASP-73	VAL-120, ILE-78, PRO-79

## Data Availability

Data is contained within the article or supplementary material.

## Supplementary Materials

The following supporting information can be downloaded at: https://www.mdpi.com/article/10.3390/ph15010086/s1, Figures S1 and S2: Photographs of Rhubarb and Rosin; Figures S3–S6: HPLC chromatograms of the isolated compounds: emodin, chrysophanol, physcion, and abietic acid; Figures S7 and S8: ^1^H-NMR spectra of the isolated emodin and abietic acid in DMSO-*d_6_*; Figures S9–S12: HRESIMS spectra of the isolated compounds: emodin, chrysophanol, physcion, and abietic acid. Table S1: Top-scoring hits retrieved from docking of AfroDb against Ddl; Table S2: Top-scoring hits retrieved from docking of AfroDb against Gyr-B; Table S3: Antibiotic susceptibility of clinical isolated E. coli MDR^a^ and MDR^b^.

## Abbreviations

Ddl: D-alanine-D-alanine ligase; Gyr-B: DNA gyrase; Δ*G*: Absolute binding free energy; MRSA: Methicillin Resistant *Staphylococcus aureus*; VRSA: Vancomycin Resistant *Staphylococcus aureus*; MDR: Multi-drug resistant; MDS: molecular dynamic simulation; PDB: protein data bank; FEP: Free Energy Perturbation; WI-38: normal human fibroblasts; RMSD: Root Mean Square Deviation; H-bonding: Hydrogen bonding; ADMET: Absorption, Distribution, Metabolism, Excretion, Toxicity properties; ATCC: American Type culture collection.
