# Supplementary material for: In Silico-Based Discovery of Natural Anthraquinones with Potential against Multidrug-Resistant E. coli
Source: Pharmaceuticals (Basel). 2022 Jan 11;15(1):86. doi: 10.3390/ph15010086 (PMC8778091; doi:10.3390/ph15010086)
Supplement: Supplementary file 1 [file pharmaceuticals-15-00086-s001.zip › pharmaceuticals-1531326-supplementary.pdf]

Supplemental Material

# In silico-based discovery of natural anthraquinones with potential against multidrug-resistant *E. coli*.

* Correspondence: Dr Ahmed M. Sayed (ahmedpharma8530@gmail.com).

Content:

Table S1. Top-scoring hits retrieved from docking of AfroDb against Ddl.

Table S2. Top-scoring hits retrieved from docking of AfroDb against Gyr-B.

Table S3. Antibiotic susceptibility of clinical isolated *E. coli* MDR[a] and MDR[b]

Figure S1 and S2. Photographs of Rhubarb and Rosin.

Figures S3-S6. HPLC chromatograms of the isolated compounds: emodin, chrysophanol, physcion, and abietic acid.

Figures S7 and S8. ^{1}H-NMR spectra of the isolated emodin and abietic acid in DMSO-d_6

Figures S9 - S12. HRESIMS spectra of the isolated compounds: emodin, chrysophanol, physcion, and abietic acid.

Methods

Table S1. Top-scoring hits retrieved from docking of AfroDb against Ddl.

No.	Name	Chemical Class	Source	Docking Score (kcal/mol)	Δ*G* (kcal/mol)	Reference
1	Protostemotinine	Alkaloid	*Stemona javanica*	-16.7	- 11.6	[1]
2	Alpinine	Alkaloid	*Delphinium alpinum*	-15.1	-11.3	[2]
3	Pisatin	Lignan	*Pisum sativum*	-14.8	-11.5	[3]
4	Salvianolic acid F	Phenolic acid	*Salvia divinorum*	-14.6	-11.1	[4]
5	Nepetoidin B	Phenolic acid	*Salvia plebeia*	-14.6	-10.8	[5]
6	Melannein	Dihydrobenzofuran	*Dalbergia melanoxylon*	-14.6	-10.5	[6]
7	Rheochrysin	Anthraquinone glycoside	*Rumex luminiastrum*	-14.5	-9.5	[7]
8	Trifoliol	Coumestan derivative	*Trifolium repens*	-14.2	-10.1	[8]
9	3'-methoxycoumestrol	Coumestan derivative	*Medicago sativa*	-14.1	-9.8	[9]
10	Sativol	Coumestan derivative	*Medicago sativa*	-14.1	-9.3	[10]
11	Coccineone B	Isoflavone	*Boerhaavia coccinea*	-13.6	-9.2	[11]
12	Anthragallol	Anthraquinone	*Galium sinaicum*	-13.4	-8.8	[12]
13	Quinizarin	Anthraquinone	*Rubia tinctorum*	-13.4	-9.1	[13]
14	Alizarin	Anthraquinone	*Rubia tinctorum*	-13.3	-8.5	[13]
15	Rhein	Anthraquinone	*Rheum palmatum*	-13.3	-8.4	[14]
16	Emodin	Anthraquinone	*Rheum palmatum*	-12.8	-9.6	[14]
17	Chrysophanol	Anthraquinone	*Rheum palmatum*	-12.6	-9.1	[14]
18	Physcion	Anthraquinone	*Rheum palmatum*	-12.6	-8.3	[14]
19	Anthrarufin	Anthraquinone	*Cassia tora*	-12.4	-8.4	[15]
20	Aloin	Anthraquinone glycoside	*Aloe species*	-12.3	-8.7	[16]
21	Helenalin	Sesquiterpene lactone	*Arnica montana*	-11.8	-8.1	[17]
22	Grosheimin	Sesquiterpene lactone	*Centaurea helenioides*	-11.5	-8.4	[17]
23	6,8,11-Epi-Desacetylmatricarin	Sesquiterpene lactone	*Matricaria chamomilla*	-10.7	-7.9	[18]
24	Mexicanin I	Sesquiterpene lactone	*Arnica montana*	-10.1	-7.3	[19]

Table S2. Top-scoring hits retrieved from docking of AfroDb against Gyr-B.

No.	Name	Chemical Class	Source	Docking Score (kcal/mol)	Δ*G* (kcal/mol)	Reference
1	Norchelerythrine	Alkaloid	*Zanthoxylum simulans*	-15.7	- 11.6	[20]
2	dihydrosanguinarine	Alkaloid	*Macleaya microcarpa*	-14.5	-10.3	[21]
3	8-Hydroxydihydrosanguinarine	Alkaloid	*Macleaya microcarpa*	-14.1	-12.5	[21]
4	6-Methoxy Dihydrosanguinarine	Alkaloid	*Macleaya microcarpa*	-13.3	-12.1	[21]
5	10-Hydroxydihydrosanguinarine	Alkaloid	*Macleaya microcarpa*	-13.3	-11.9	[21]
6	Sanguinarine	Alkaloid	*Macleaya microcarpa*	-13.1		[21]
7	Sophoranochromene	Flavonoid	*Sophora subprostrata*	-12.8	-11.3	[22]
8	Ohioensin G	Flavonoid	*Polytrichastrum alpinum*	-12.6	-10.2	[23]
9	Ohioensin F	Flavonoid	*Polytrichastrum alpinum*	-12.4	-10.8	[23]
10	Ugaxanthone	Xanthone	*Symphonia globulifera*	-12.3	-6.6	[24]
11	Toxyloxanthone C	Xanthone	*Cudrania cochinchinensis*	-12.3	-9.5	[25]
12	Hyperxanthone E	Xanthone	*Hypericum calycinum*	-12.2	-6.9	[26]
13	Triptobenzene H	Diterpene	*Tripterygium wilfordii*	-11.7	-9.2	[27]
14	12-Methyl-5-dehydrohorminone	Diterpene	*Salvia multicaulis*	-11.6	-9.1	[28]
15	Merogedunin	Diterpene	*Cedrela odorata*	-11.3	-8.9	[29]
16	Abietic acid	Diterpene	*Pimenta racemosa*	-11.1	-8.8	[30]
17	Pimaric acid	Diterpene	*Pimenta racemosa*	-11.1	-8.3	[30]
18	Neoabietic acid	Diterpene	*Pimenta racemosa*	-11.0	-8.7	[30]
19	Hugorosenone	Diterpene	*Hugonia casteneifolia*	-10.8	-8.5	[31]
20	Totaradiol	Diterpene	*Thuja plicata*	-10.8	-8.4	[32]
21	Hinokiol	Diterpene	*Rosmarinus officinalis*	-10.7	-8.3	[33]
22	Dehydroabietic acid	Diterpene	*Pimenta racemosa*	-10.6	-9.1	[33]
23	Emodin	Anthraquinone	*Rheum palmatum*	-10.4	-9.4	[14]
24	Chrysophanol	Anthraquinone	*Rheum palmatum*	-10.4	-9.3	[14]
25	Rhein	Anthraquinone	*Rheum palmatum*	-10.4	-9.1	[14]
26	Dicentrine	Alkaloid	*Stephania epigeae*	-10.2	-6.5	[34]
27	Papaverine	Alkaloid	*Papaver somniferum*	-10.1	-6.2	[34]

Table S3. Antibiotic susceptibility of clinical isolated *E. coli* MDR[a] and MDR[b]

Clinical Strain	Antibiotics	MIC (µg/mL)
E. coli MDR[a]	Ampicillin	>64
	Ceftazidime	32
	Ceftazolin	32
	Cefotaxime	32
	Levofloxacin	16
	Ciprofloxacin	16
E. coli MDR[b]	Ampicillin	>64
	Ceftazidime	16
	Ceftazolin	32
	Cefotaxime	64
	Azithromycin	16
	Amikacin	8

[a] and [b] are multidrug-resistant *E. coli* clinical isolates.

These susceptibility testing results are not published data. The testing was carried out by the microbiology unit in Beni Suef general hospital, Beni Suef, Egypt using the same procedure stated in the methods section in the main manuscript.

Methods

Molecular Dynamics Simulation

Desmond v. 2.2 software was used for performing MDS experiments [35, 36], while NMAD 2.14 was used to run MDS required for FEP-based ΔG estimation [38]. For simulations produced by Desmond, the OPLS 2005 force field was applied. Protein systems were built using the System Builder option, where the protein structure was checked for any missing hydrogens, the protonation states of the amino acid residues were set (pH = 7.4), and the co-crystalized water molecules were removed. Thereafter, the whole structure was embedded in an orthorhombic box of TIP3P water [37] together with 0.15 M Na^+ and Cl^- ions in 20 Å solvent buffer. Afterward, the prepared systems were energy minimized and equilibrated for 10 ns. Subsequently the production simulation was set to be 50 ns. All simulations (equilibration and production) were set to be run under NPT ensembles [35, 36] at temperature of 300 K. For protein-ligand complexes, the top-scoring poses were used as a starting points for simulation. Desmond software automatically parameterizes inputted ligands during the system building step according to the OPLS force field. For simulations performed by NAMD [38], the protein structures were built and optimized by using the QwikMD toolkit of the VMD software [38, 39]. The parameters and topologies of the compounds were calculated either using the Charmm27 force field with the online software Ligand Reader and Modeler (http://www.charmm-gui. org/?doc=input/ligandrm, accessed on 16 April

2021) [39] or using the VMD plugin Force Field Toolkit (ffTK). Afterward, the generated parameters and topology files were loaded to VMD to readily read the protein–ligand complexes without errors and then conduct the simulation step.

Binding Free Energy Calculations

Binding free energy calculations (Δ*G*) were performed using the free energy perturbation (FEP) method [39]. This method was described in detail in the recent article by Kim and coworkers [38]. Briefly, this method calculates the binding free energy $\Delta G_{binding}$ according to the following equation: $\Delta G_{binding} = \Delta G_{Complex} - \Delta G_{Ligand}$. The value of each Δ*G* is estimated from a separate simulation using NAMD software. Interestingly, all input files required for simulation by NAMD can be papered by using the online website Charmm-GUI (https://charmm-gui.org/?doc=input/afes.abinding, accessed on 22 June 2021). Subsequently, we can use these files in NAMD to produce the required simulations using the FEP calculation function in NAMD. The equilibration (5 ns long) was achieved in the NPT ensemble at 300 K and 1 atm (1.01325 bar) with Langevin piston pressure (for "Complex" and "Ligand") in the presence of the TIP3P water model [37]. Then, 10 ns FEP simulations were performed for each compound, and the last 5 ns of the free energy values was measured for the final free energy values [39]. Finally, the generated trajectories were visualized and analyzed using VMD software [38, 39].

Isolation of the Rhubarb anthraquinones

Dry roots of Rhubarb (*Rheum officinale* Bail; Voucher number: RO-102-NUP) (Figure S1) (500 g) were washed, triturated, and exhaustively macerated in MeOH (1000 mL) at room temperature for 24 h. All the MeOH extracts were combined, filtered off, and concentered on a rotary evaporation to give 390 g a mixture of anthraquinones. This crude extract was then exhaustively fractionated with DCM (500 mL X 3) yielding of (after solvent evaporation) 41 g (14 %) of brown solid crude anthraquinones.

Purification process was performed by silica gel CC (glass columns, 60 x 600 mm) using a binary mobile phase combination of DCM: EtOAc with a gradient of 0-15 % to yield emodin (75 mg), chrysophanol (44 mg), and physcion (36 mg). The eluted compounds were analyzed by TLC using DCM : Hex (9:1, v/v) as a mobile phase and combined according to reference of the retention time (R_f) values of the standard reference compounds [40].

Isolation of abietic acid from Rosin

Rosin (100 g) is placed in a 1 L round-bottomed flask, ethanol (500 mL) and concentrated HCl (15 mL) are added and the solution is refluxed for 2.5 hours. Ethanol and water are then removed in vacuo. The remaining dark orange residue is dissolved in diethyl ether (200 mL), washed with water to remove any HCl and dried over Na_2SO_4 (80 g). The Na_2SO_4 is removed by filtration and the ether by distillation in vacuo. The remaining residue is warmed in a hot water bath and poured on to a clean ceramic plate on a laboratory bench to cool to a brittle mass.

This mass is broken, transferred into a 500 mL Erlenmeyer flask and dissolved in methanol (150 mL). At incipient boiling of the solution, ammonia solution (40 mL) is added slowly with vigorous stirring for 1 hour.

The flask was allowed to stand overnight for cooling to ambient temperature. Crystals separate. The flask is then immersed in an ice bath, the mixture is stirred to increase crystallization and finally the crystals of the crude ammonium abietate are filtered by suction (76.5 g)

All of this salt were placed in a 1 L Erlenmeyer flask and dissolved in hot ethanol (200 mL). After cooling, glacial acetic acid (10 g) is added in one portion with vigorous stirring. Water (180 mL) is added slowly with vigorous agitation until the first crystals of abietic acid appear (55 g) [40].

To check for the isolated compounds' purity, they were subjected to LC-HRESIMS analysis. Briefly, the isolated compounds were dissolved in 50% aqueous acetonitrile to prepare stock solutions (1 mg mL^{-1}). Then, 1 mL was filtered through a 0.22 µm membrane filter before introduction to the HPLC system. Chromatographic separation was performed on a BEH C18 column (2.1 × 100 mm, 1.7 µm particle size; Waters, Milford, CT, USA) with a guard column (2.1 × 5 mm, 1.7 µm particle size) and a linear solvent gradient of 0–100% eluent B at a flow rate of 0.3 mL min^{-1} over 6 min, using 0.1% formic acid in water (v/v) as solvent A and acetonitrile as solvent B. The injection volume was 2 µL and the column temperature was 40 °C [41]. High-resolution mass spectral data were obtained from a Thermo Instruments MS system (LTQ XL/LTQ Orbitrap Discovery) coupled to a Thermo Instruments HPLC system (Accela PDA detector, Accela PDA autosampler and Accela Pump). The following conditions were used: capillary voltage 45 V, capillary temperature 260°C, auxiliary gas flow rate 10–20 arbitrary units, sheath gas flow rate 40–50 arbitrary units, spray voltage 45 kV, mass range 100–2000 amu (maximum resolution 30 000). Additionally, the ^{1}H-NMR spectra of emodin and abietic acid were obtained in DMSD-d_6 on a Bruker-400 spectrometer (400 MHz for ^{1}H). All of the chemical shifts (δ) are

given in ppm units with reference to TMS as an internal standard, and coupling constants (J) are reported in Hz.

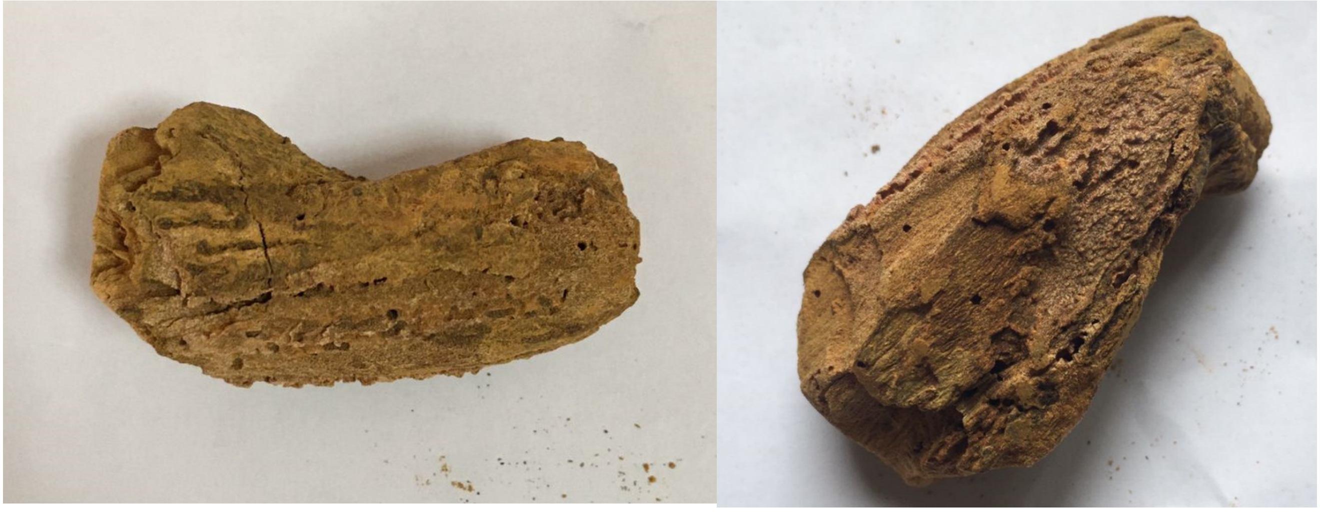

Figure S1. Photographs of Rhubarb (*Rheum officinal* Bail; Voucher number: RO-102-NUP).

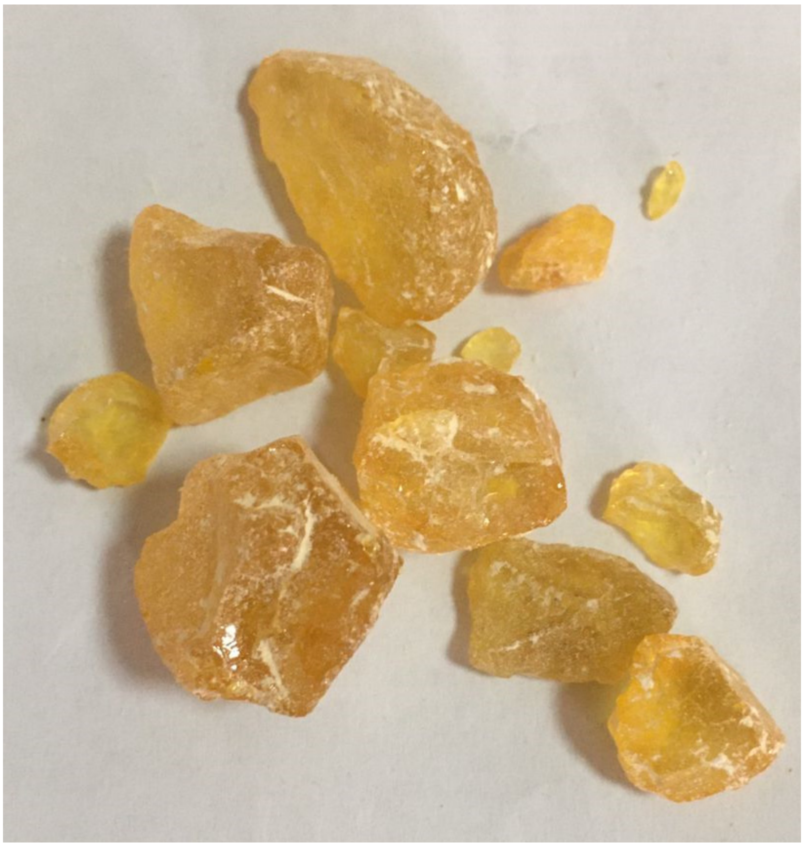

Figure S2. Photograph of Colophony (Rosin; Voucher number: CR-34-NUP).

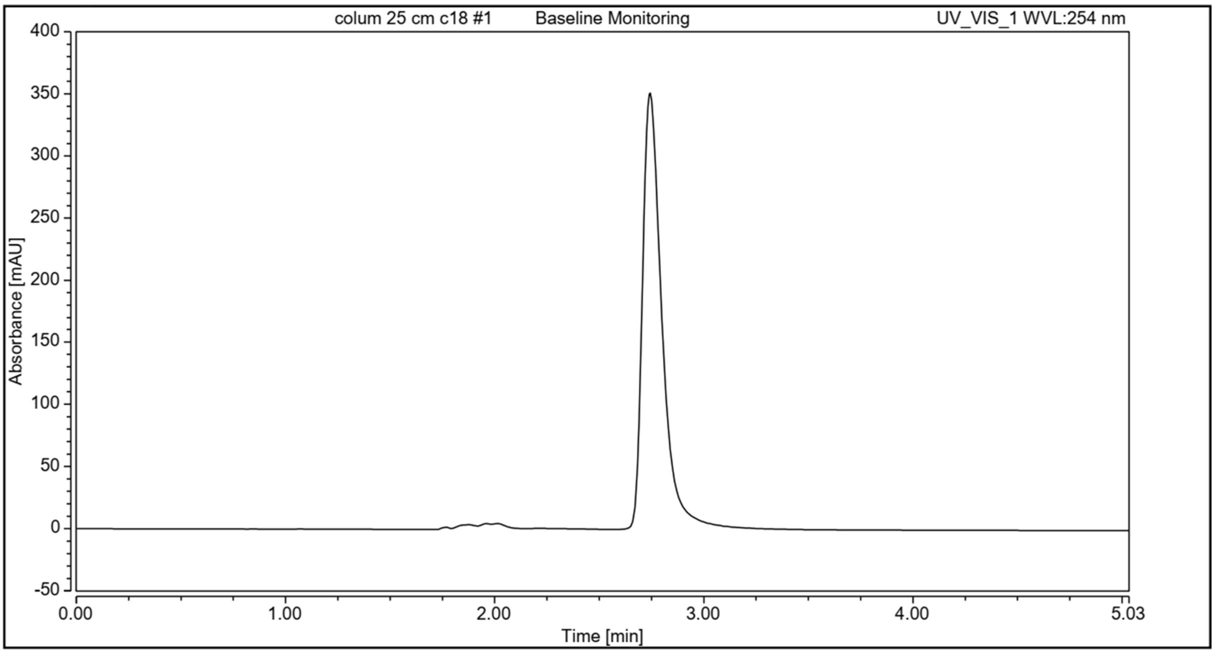

Figure S3. HPLC chromatogram of emodin.

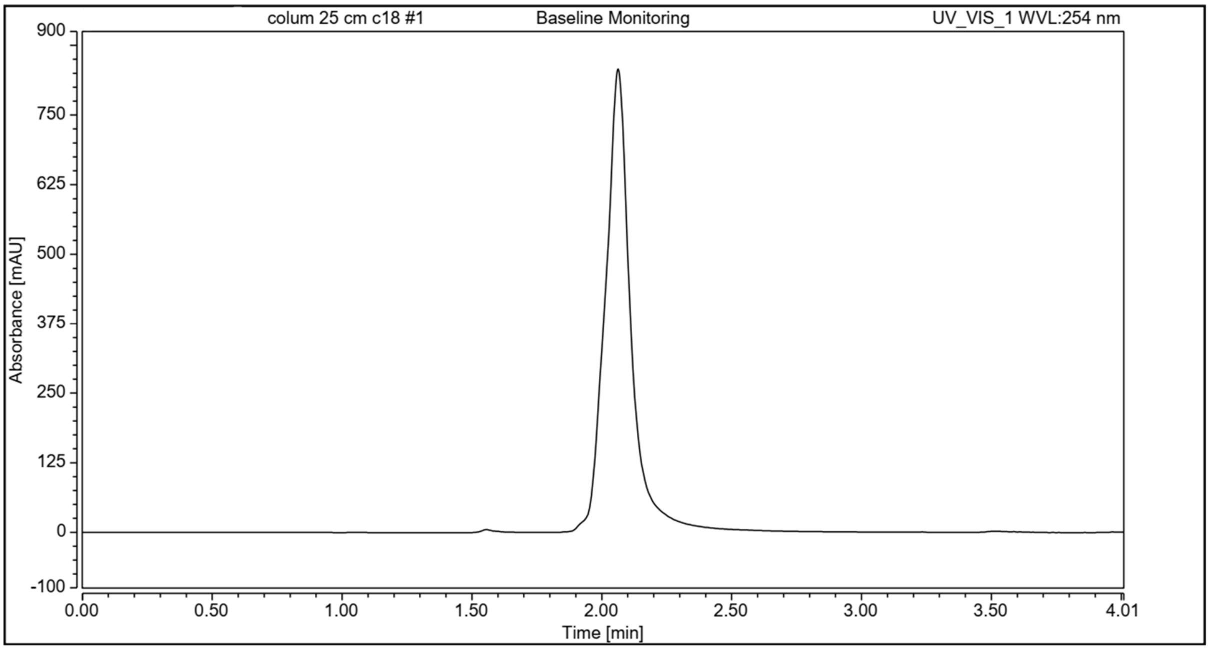

Figure S4. HPLC chromatogram of chrysophanol.

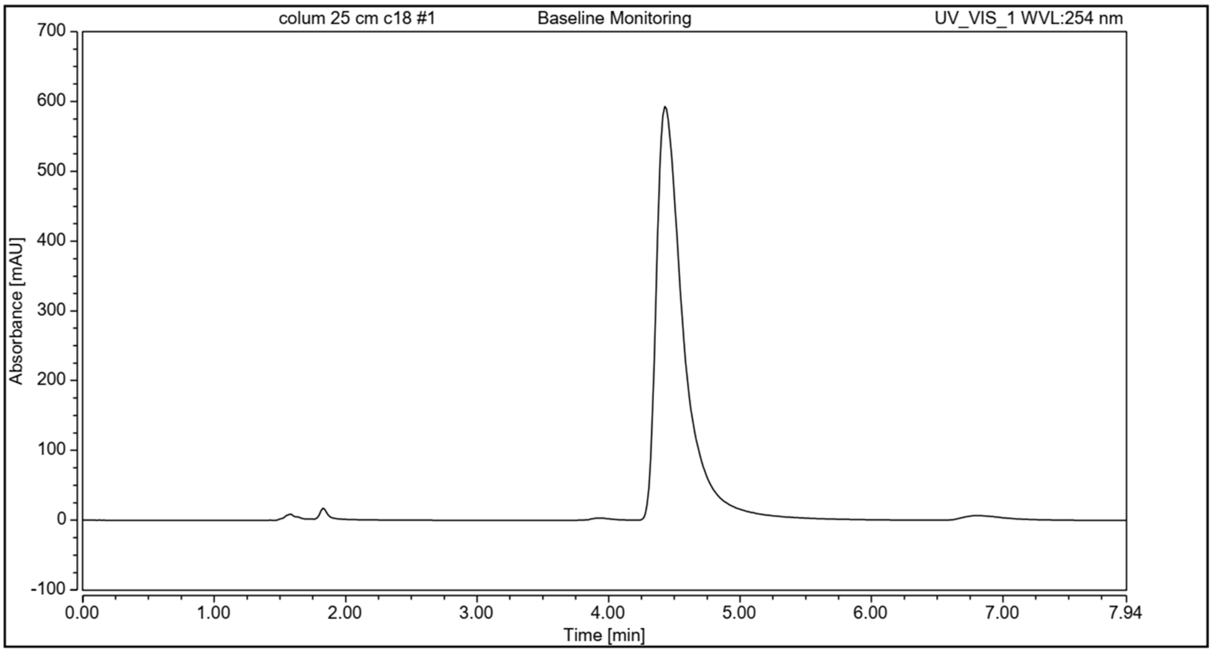

Figure S5. HPLC chromatogram of physcion.

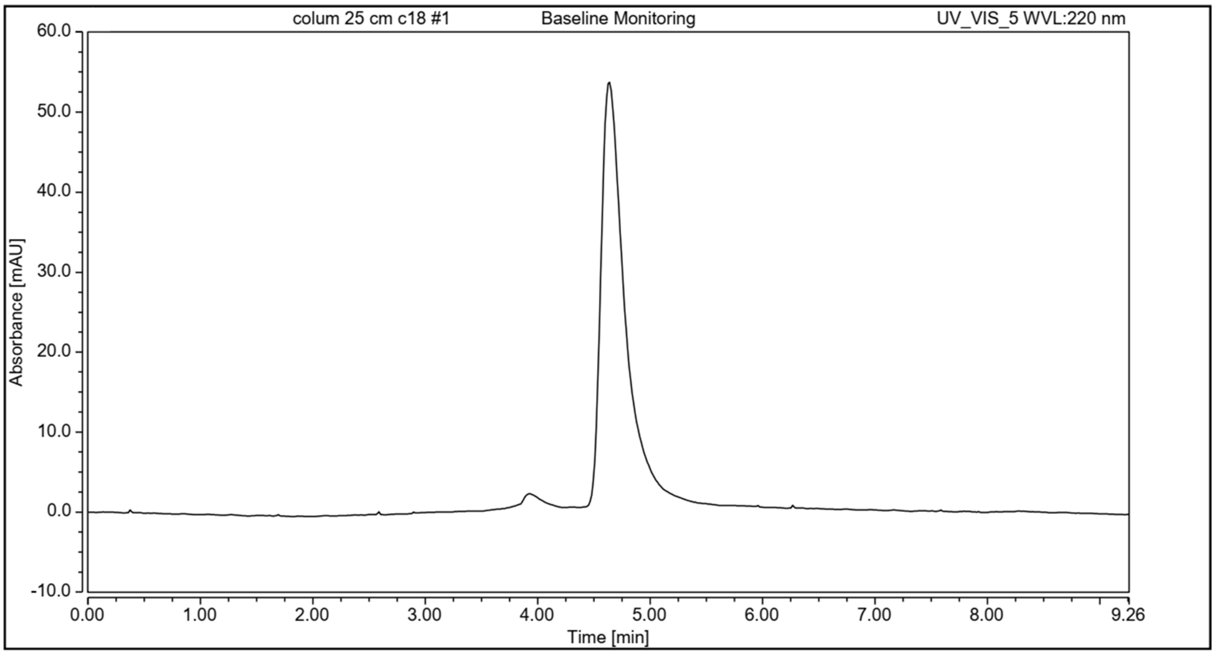

Figure S6. HPLC chromatogram of abietic acid.

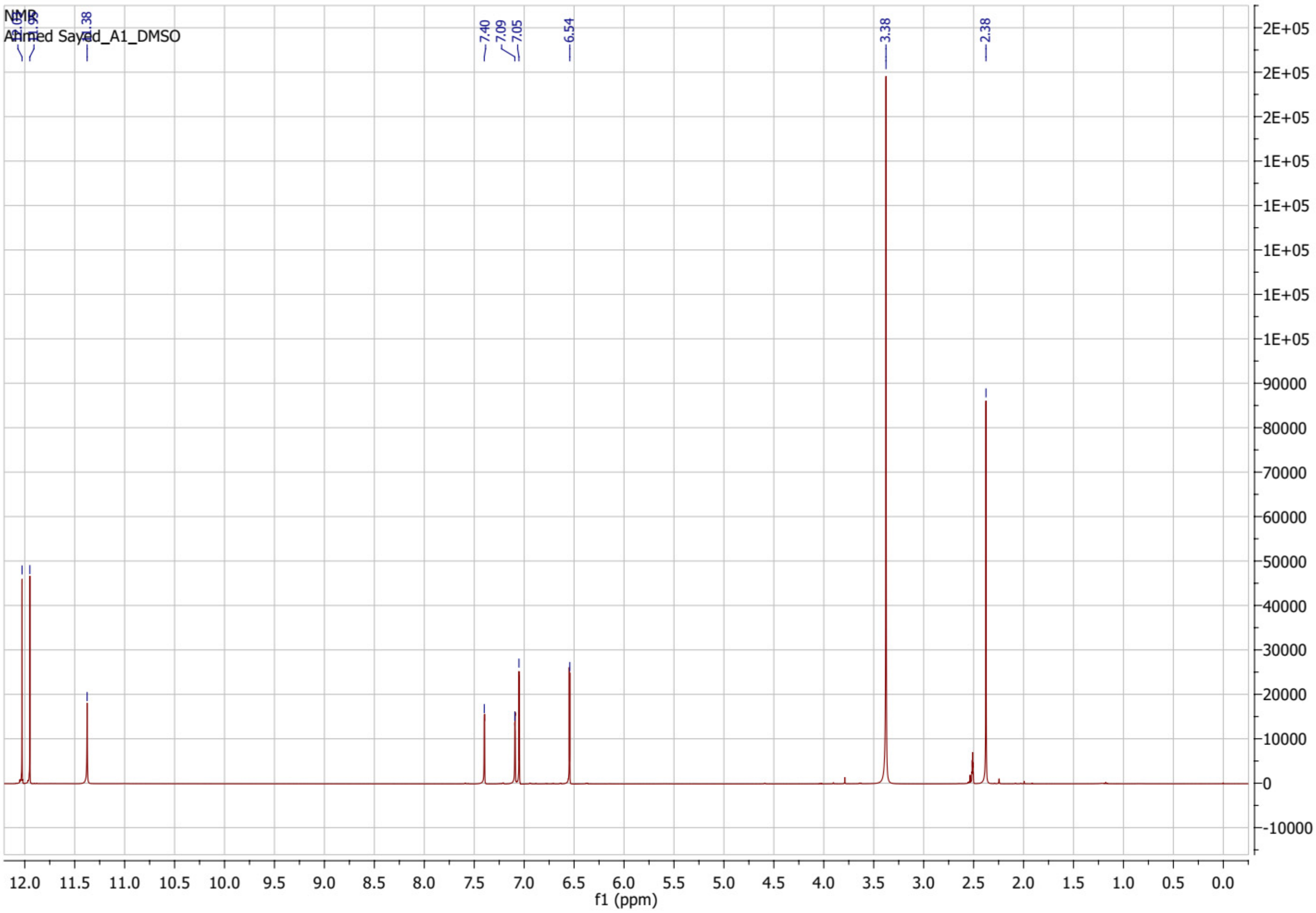

Figure S7. ^{1}H-NMR spectrum of the isolated emodin in DMSO-d_6

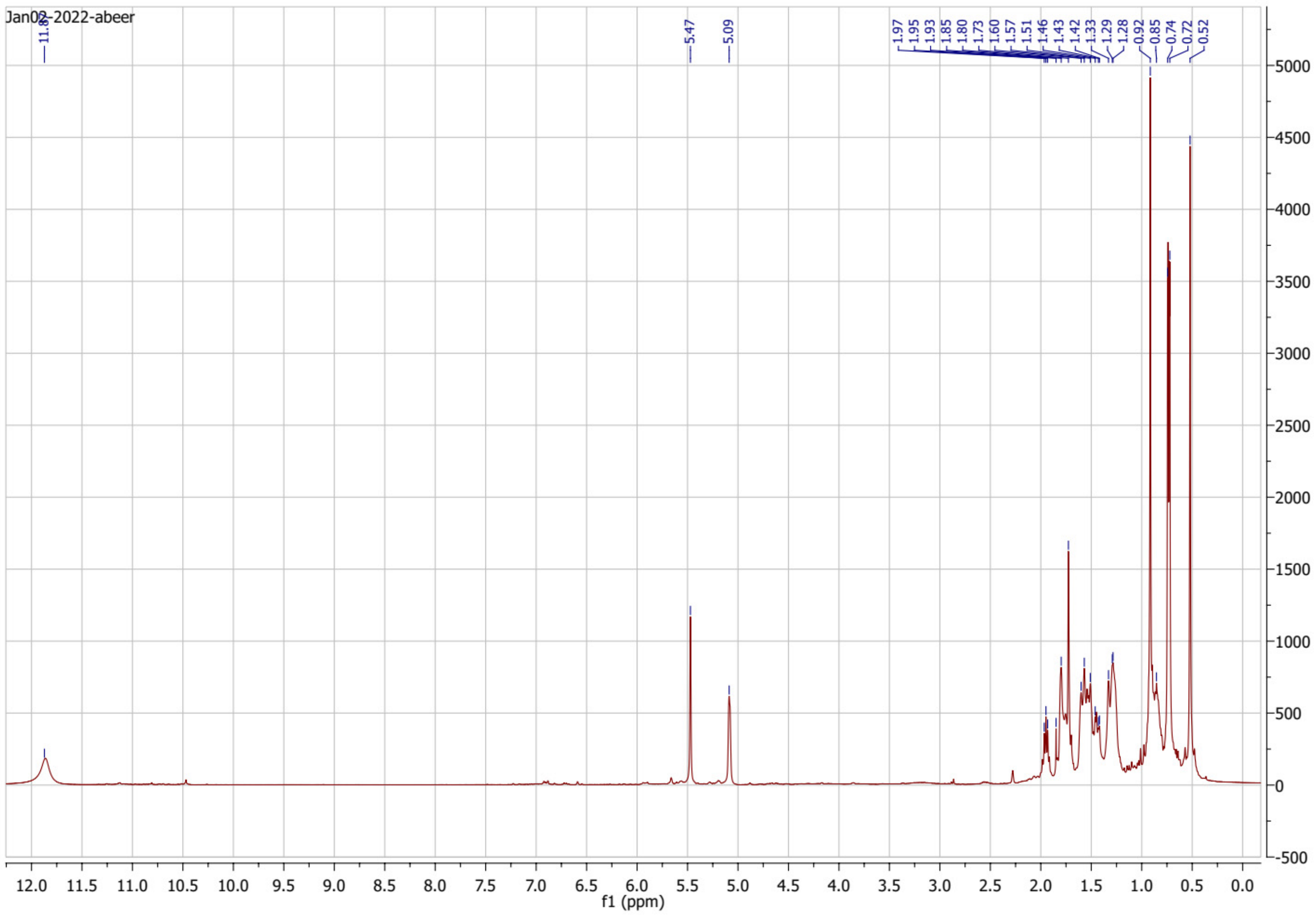

Figure S8. ^{1}H-NMR spectrum of the isolated abietic acid in DMSO-d_6

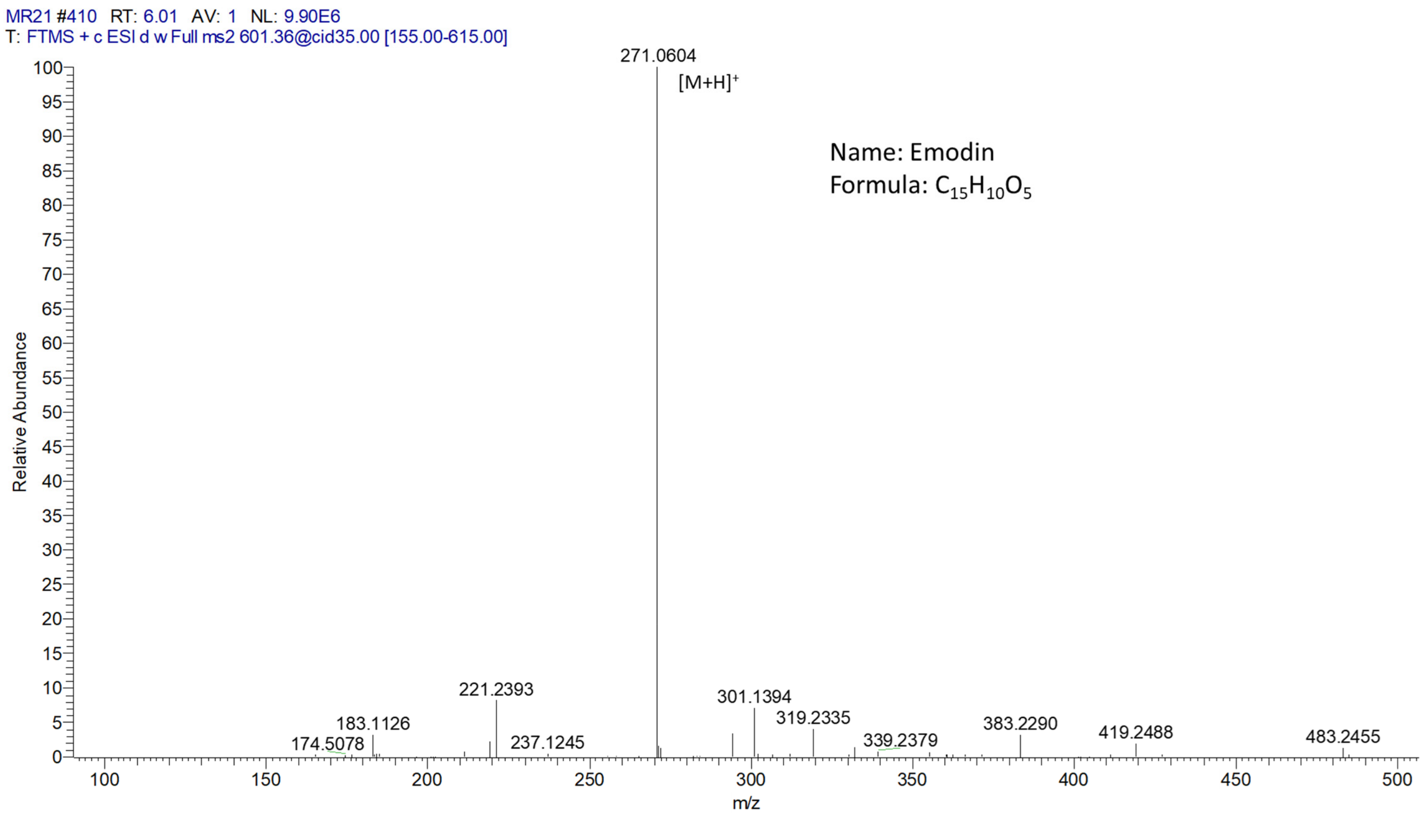

Figure S9. HRESIMS spectrum of emodin.

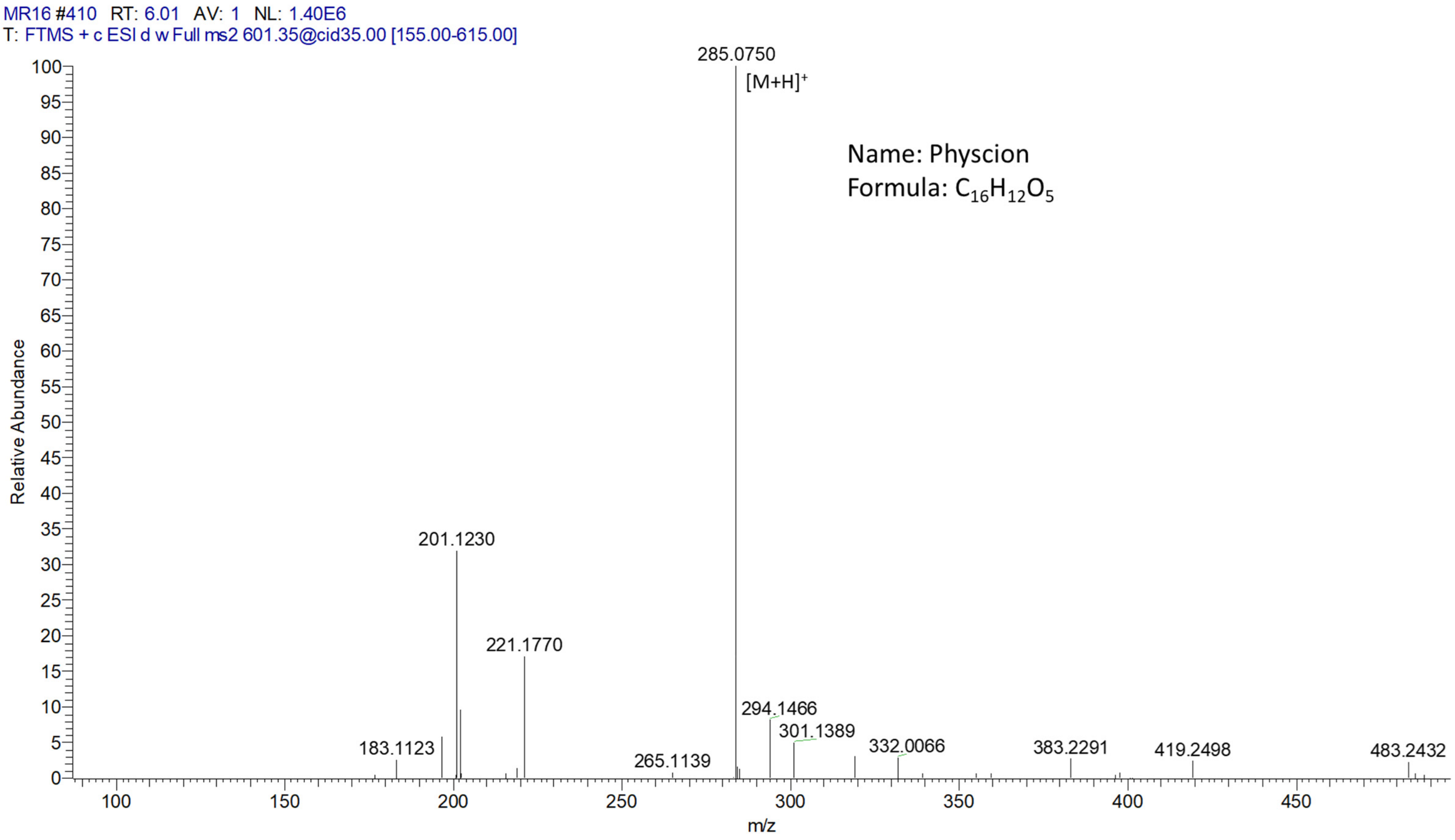

Figure S10. HRESIMS spectrum of Physcion.

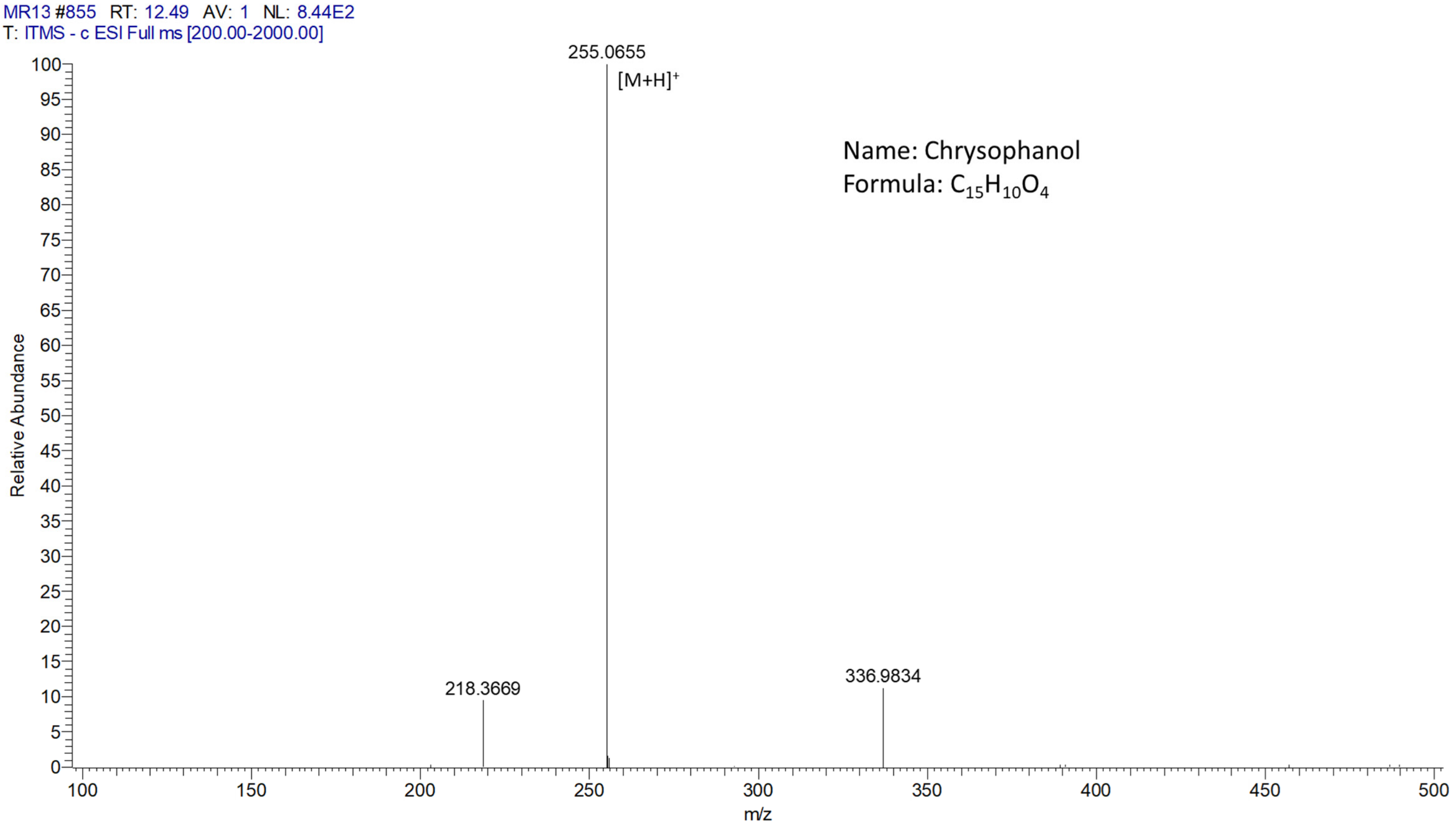

Figure S11. HRESIMS spectrum of chrysophanol.

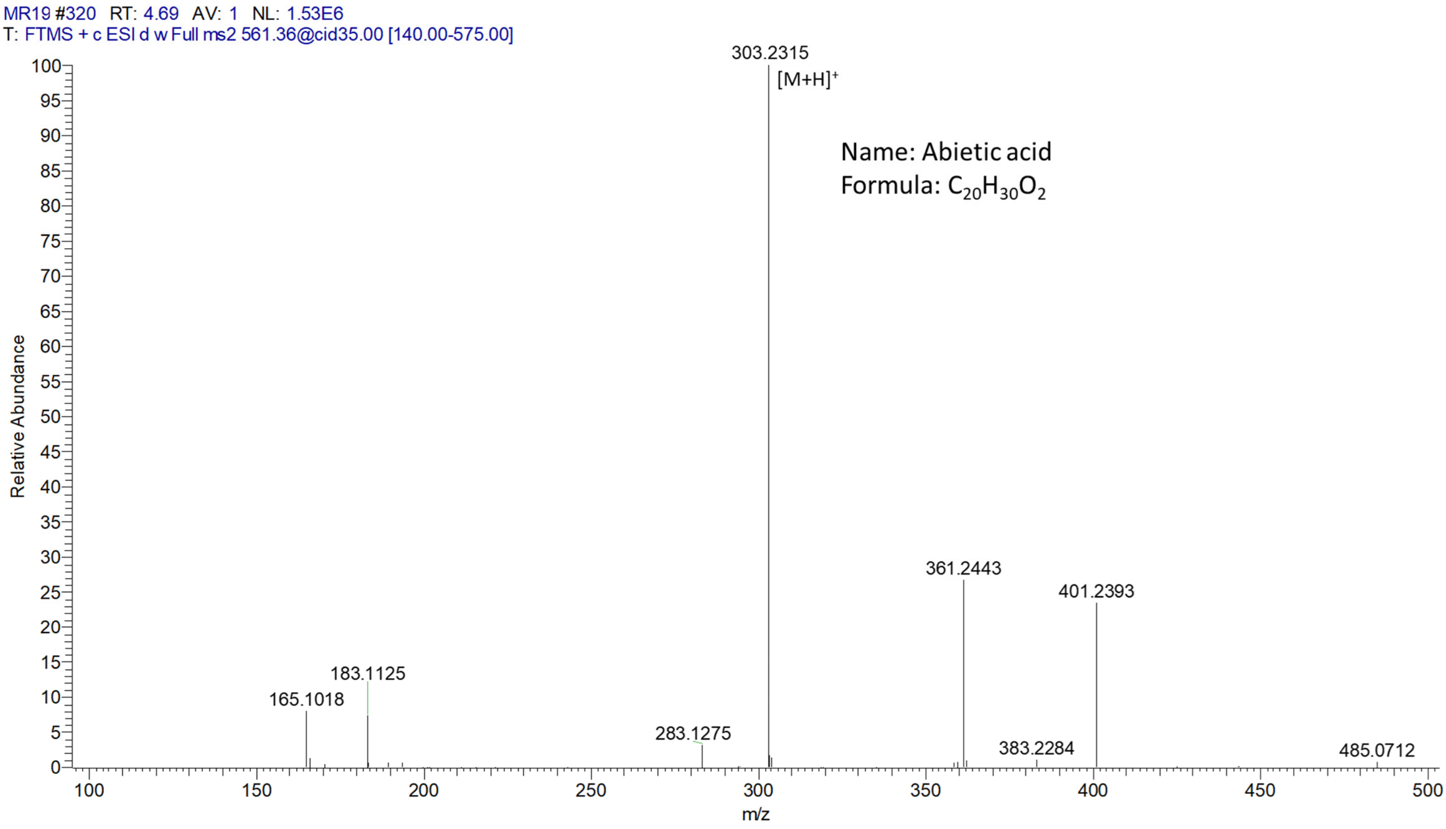

Figure S12. HRESIMS spectrum of abietic acid.

References

1. Hosoya, T., Yamasaki, F., Nakata, A., Rahman, A., Kusumawati, I., Zaini, N. C., & Morita, H. (2011). Inhibitors of nitric oxide production from Stemona javanica. *Planta medica*, *77*(03), 256-258.

2. Khairitdinova, E. D., Tsyrlina, E. M., Spirikhin, L. V., Fedorov, N. I., & Yunusov, M. S. (2005). Alpinine, a new norditerpene alkaloid from Delphinium alpinum. *Chemistry of natural compounds*, *41*(5), 575-577.

3. Selim, S., Sanssené, J., Rossard, S., & Courtois, J. (2017). Systemic induction of the defensin and phytoalexin pisatin pathways in pea (Pisum sativum) against Aphanomyces euteiches by acetylated and nonacetylated oligogalacturonides. *Molecules*, *22*(6), 1017.

4. Dalla, V., & Cotelle, P. (1999). The total synthesis of salvianolic acid F. *Tetrahedron*, *55*(22), 6923-6930.

5. Kim, M., Kim, J. Y., Yang, H. S., Choe, J. S., & Hwang, I. G. (2021). Nepetoidin B from Salvia plebeia R. Br. Inhibits Inflammation by Modulating the NF-κB and Nrf2/HO-1 Signaling Pathways in Macrophage Cells. *Antioxidants*, *10*(8), 1208.

6. Donnelly, B. J., Donnelly, D. M. X., O'Sullivan, A. M., & Prendergast, J. P. (1969). Dalbergia species—VII: The isolation and structure of melanoxin a new dihydrobenzofuran from Dalbergia melanoxylon guill. and perr.(leguminoseae). *Tetrahedron*, *25*(18), 4409-4414.

7. Abd el-Fattah, H., Gohar, A., El-Dahmy, S., & Hubaishi, A. (1994). Phytochemical investigation of Rumex luminiastrum. *Acta Pharmaceutica Hungarica*, *64*(3), 83-85.

8. Livingston, A. L., Bickoff, E. M., Lundin, R. E., & Jurd, L. (1964). Trifoliol, a new coumestan from ladino clover. *Tetrahedron*, *20*(8), 1963-1970.

9. Bickoff, E. M., Spencer, R. R., Knuckles, B. E., & Lundin, R. E. (1966). 3'-Methoxycoumestrol from alfalfa: isolation and characterization. *Journal of Agricultural and Food Chemistry*, *14*(5), 444-446.

10. Spencer, R. R., Bickoff, E. M., Lundin, R. E., & Knuckles, B. E. (1966). New Alfalfa Compounds, Lucernol and Sativol, Two New Coumestans from Alfalfa (Medicago sativa). *Journal of Agricultural and Food Chemistry*, *14*(2), 162-165.

11. Ferrari, F., Messana, I., & Ana, A. E. G. S. (1991). Two new isoflavonoids from Boerhaavia, coccinea. *Journal of natural products*, *54*(2), 597-598.

12. El-Gamal, A. A., Takeya, K., Itokawa, H., Halim, A. F., Amer, M. M., Saad, H. E., & Awad, S. A. (1996). Anthraquinones from the polar fractions of Galium sinaicum. *Phytochemistry*, *42*(4), 1149-1155.

13. Derksen, G. C., Niederländer, H. A., & van Beek, T. A. (2002). Analysis of anthraquinones in Rubia tinctorum L. by liquid chromatography coupled with diode-array UV and mass spectrometric detection. *Journal of chromatography A*, *978*(1-2), 119-127.

14. Hörhammer, L., Wagner, H., & Köhler, I. (1959). Neue Untersuchungen über die Inhaltsstoffe von Rheum palmatum L. 1. Mitteilung: Zur Analytik des Rheins. *Archiv der Pharmazie*, *292*(11), 591-601.

15. Lee, H. S. (2003). Inhibitory effects of quinizarin isolated from Cassia tora seeds against human intestinal bacteria and aflatoxin $ B_1 $ biotransformation. *Journal of microbiology and biotechnology*, *13*(4), 529-536.

16. Reynolds, T. (Ed.). (2004). *Aloes: the genus Aloe*. CRC press.

17. Perry, N. B., Burgess, E. J., Guitián, M. A. R., Franco, R. R., Mosquera, E. L., Smallfield, B. M., ... & Littlejohn, R. P. (2009). Sesquiterpene lactones in Arnica montana: helenalin and dihydrohelenalin chemotypes in Spain. *Planta medica*, *75*(06), 660-666.

18. Čekan, Z., Prochazka, V., Herout, V., & Šorm, F. (1959). On terpenes. CI. Isolation and constitution of matricarin, another guaianolide from camomile (Matricaria chamomilla L.). *Collection of Czechoslovak Chemical Communications*, *24*(5), 1554-1557.

19. Picman, A. K. (1986). Biological activities of sesquiterpene lactones. *Biochemical systematics and Ecology*, *14*(3), 255-281.

20. Chen, I. S., Wu, S. J., Tsai, I. L., Wu, T. S., Pezzuto, J. M., Lu, M. C., ... & Teng, C. M. (1994). Chemical and bioactive constituents from Zanthoxylum simulans. *Journal of Natural Products*, *57*(9), 1206-1211.

21. Yao, J. Y., Zhou, Z. M., Li, X. L., Yin, W. L., Ru, H. S., Pan, X. Y., ... & Shen, J. Y. (2011). Antiparasitic efficacy of dihydrosanguinarine and dihydrochelerythrine from Macleaya microcarpa against Ichthyophthirius multifiliis in richadsin (Squaliobarbus curriculus). *Veterinary Parasitology*, *183*(1-2), 8-13.

22. Chang-Ming, H. E., Cheng, Z. H., & Dao-Feng, C. H. E. N. (2013). Qualitative and quantitative analysis of flavonoids in Sophora tonkinensis by LC/MS and HPLC. *Chinese journal of natural medicines*, *11*(6), 690-698.

23. Seo, C., Choi, Y. H., Sohn, J. H., Ahn, J. S., Yim, J. H., Lee, H. K., & Oh, H. (2008). Ohioensins F and G: Protein tyrosine phosphatase 1B inhibitory benzonaphthoxanthenones from the Antarctic moss Polytrichastrum alpinum. *Bioorganic & medicinal chemistry letters*, *18*(2), 772-775.

24. Locksley, H. D., Moore, I., & Scheinmann, F. (1966). Extractives from guttiferae. Part IV. Isolation and structure of ugaxanthone and mbarraxanthone from Symphonia globulifera L. *Journal of the Chemical Society C: Organic*, 2265-2269.

25. Fukai, T., Yonekawa, M., Hou, A. J., Nomura, T., Sun, H. D., & Uno, J. (2003). Antifungal agents from the roots of cudrania c ochinchinensis against candida, cryptococcus, and aspergillus species. *Journal of natural products*, *66*(8), 1118-1120.

26. Gaid, M. M., Sircar, D., Müller, A., Beuerle, T., Liu, B., Ernst, L., ... & Beerhues, L. (2012). Cinnamate: CoA ligase initiates the biosynthesis of a benzoate-derived xanthone phytoalexin in Hypericum calycinum cell cultures. *Plant physiology*, *160*(3), 1267-1280.

27. Duan, H., Takaishi, Y., Momota, H., Ohmoto, Y., Taki, T., Jia, Y., & Li, D. (1999). Immunosuppressive diterpenoids from Tripterygium wilfordii. *Journal of natural products*, *62*(11), 1522-1525.

28. Ulubelen, A., Topcu, G., & Johansson, C. B. (1997). Norditerpenoids and Diterpenoids from Salvia m ulticaulis with Antituberculous Activity. *Journal of Natural Products*, *60*(12), 1275-1280.

29. MacKinnon, S., Durst, T., Arnason, J. T., Angerhofer, C., Pezzuto, J., Sanchez-Vindas, P. E., ... & Gbeassor, M. (1997). Antimalarial activity of tropical Meliaceae extracts and gedunin derivatives. *Journal of Natural Products*, *60*(4), 336-341.

30. Fernandez, M. A., Tornos, M. P., Garcia, M. D., De las Heras, B., Villar, A. M., & Saenz, M. T. (2001). Anti-inflammatory activity of abietic acid, a diterpene isolated from Pimenta racemosa var. grissea. *Journal of Pharmacy and Pharmacology*, *53*(6), 867-872.

31. Mdee, L. K., Waibel, R., Nkunya, M. H., Jonker, S. A., & Achenbach, H. (1998). Rosane diterpenes and bis-dinorditerpenes from Hugonia casteneifolia. *Phytochemistry*, *49*(4), 1107-1113.

32. Sharp, H., Latif, Z., Bartholomew, B., Bright, C., Jones, C. D., Sarker, S. D., & Nash, R. J. (2001). Totarol, totaradiol and ferruginol: three diterpenes from Thuja plicata (Cupressaceae). *Biochemical systematics and ecology*, *29*(2), 215-217.

33. Cantrell, C. L., Richheimer, S. L., Nicholas, G. M., Schmidt, B. K., & Bailey, D. T. (2005). s eco-Hinokiol, a New Abietane Diterpenoid from Rosmarinus officinalis. *Journal of natural products*, *68*(1), 98-100.

34. Kiger, R. W. (1973). Sectional Nomenclature in Papaver L. *Taxon*, 579-582.

35. Bowers, K.J.; Chow, D.E.; Xu, H.; Dror, R.O.; Eastwood, M.P.; Gregersen, B.A.; Klepeis, J.L.; Kolossvary, I.; Moraes, M.A.; Sacerdoti, F.D.; et al. Scalable algorithms for molecular dynamics simulations on commodity clusters. In Proceedings of the SC'06: Proceedings of the 2006 ACM/IEEE Conference on Supercomputing, Tampa, FL, USA, 11–17 November 2006; IEEE: New York, NY, USA, 2006; p. 43.

36. Release, S. 3: Desmond Molecular Dynamics System, DE Shaw Research, New York, NY, 2017; Maestro-Desmond Interoperability Tools, Schrödinger: New York, NY, USA, 2017.

37. Mark, P., & Nilsson, L. (2001). Structure and dynamics of the TIP3P, SPC, and SPC/E water models at 298 K. *The Journal of Physical Chemistry A*, *105*(43), 9954-9960.

38. Phillips, J.C.; Braun, R.; Wang, W.; Gumbart, J.; Tajkhorshid, E.; Villa, E.; Chipot, C.; Skeel, R.D.; Kalé, L.; Schulten, K. Scalable molecular dynamics with NAMD. J. Comput. Chem. 2005, 26, 1781–1802.

39. Kim, S.; Oshima, H.; Zhang, H.; Kern, N.R.; Re, S.; Lee, J.; Rous, B.; Sugita, Y.; Jiang, W.; Im, W. CHARMM-GUI free energy calculator for absolute and relative ligand solvation and binding free energy simulations. J. Chem. Theory Comput. 2020, 16, 7207–7218.

40. Berger, S., & Sicker, D. (2009). *Classics in spectroscopy: isolation and structure elucidation of natural products*. John Wiley & Sons.

41. Elhawary, S. S., Sayed, A. M., Issa, M. Y., Ebrahim, H. S., Alaaeldin, R., Abd El-Kader, E. M., & Abdelmohsen, U. R. (2021). Anti-Alzheimer Chemical Constituents of Morus macroura Miq.: Chemical Profiling, In silico, and In vitro Investigations. *Food & Function*.
